# UV-induced reactive oxygen species and transcriptional control of 3-deoxyanthocyanidin biosynthesis in black sorghum pericarp

**DOI:** 10.3389/fpls.2024.1451215

**Published:** 2024-10-07

**Authors:** Brooklyn Schumaker, Lauren Mortensen, Robert R. Klein, Sabyasachi Mandal, Linda Dykes, Nicholas Gladman, William L. Rooney, Byron Burson, Patricia E. Klein

**Affiliations:** ^1^ Department of Horticultural Sciences, Texas A&M University, College Station, TX, United States; ^2^ USDA-ARS, Southern Plains Agricultural Research Center, College Station, TX, United States; ^3^ Department of Biology, Texas A&M University, College Station, TX, United States; ^4^ USDA-ARS, Cereal Crops Research Unit, Edward T. Schafer Agricultural Research Unit, Fargo, ND, United States; ^5^ USDA-ARS, Robert W. Holley Center for Agriculture and Health, Cornell University, Ithaca, NY, United States; ^6^ Cold Spring Harbor Laboratory, Cold Spring Harbor, NY, United States; ^7^ Department of Soil and Crop Sciences, Texas A&M University, College Station, TX, United States

**Keywords:** 3-deoxyanthocyanidin, sorghum, pericarp, transcriptomics, gene co-expression network, UV light, ROS

## Abstract

Black pericarp sorghum has notable value due to the biosynthesis of 3-deoxyanthocyanidins (3-DOAs), a rare class of bioactive polyphenols valued as antioxidant food additives and as bioactive compounds with cytotoxicity to human cancer cells. A metabolic and transcriptomic study was conducted to ascertain the cellular events leading to the activation of 3-DOA biosynthesis in black sorghum pericarp. Prolonged exposure of pericarp during grain maturation to high-fluence ultraviolet (UV) light resulted in elevated levels of reactive oxygen species (ROS) and the activation of 3-DOA biosynthesis in pericarp tissues. In conjunction with 3-DOA biosynthesis was the transcriptional activation of specific family members of early and late flavonoid biosynthesis pathway genes as well as the downstream activation of defense-related pathways. Promoter analysis of genes highly correlated with 3-DOA biosynthesis in black pericarp were enriched in MYB and HHO5/ARR-B motifs. Light microscopy studies of black pericarp tissues suggest that 3-DOAs are predominantly localized in the epicarp and are associated with the cell wall. A working model of UV-induced 3-DOA biosynthesis in black pericarp is proposed that shares features of plant immunity associated with pathogen attack or mechanical wounding. The present model depicts ROS accumulation, the transcriptional activation of receptor kinases and transcription factors (TFs) including NAC, WRKY, bHLH, AP2, and C2H2 Zinc finger domain. This study identified key biosynthetic and regulatory genes of 3-DOA accumulation in black pericarp and provided a deeper understanding of the gene networks and cellular events controlling this tissue-and genotype-specific trait.

## 1 Introduction

Flavonoids are secondary metabolites that serve essential functions in plant vegetative and reproductive structures ranging from the regulation of cell growth, pollinator attractors, participating in plant-microbe and plant-herbivore interactions, and as defense mechanisms to abiotic and biotic stresses (Dias et al., 2021). Secondary metabolites are critical not only for plant vegetative and reproductive development, but they also represent a wide array of highly valued phytochemicals for human health. Cereal polyphenols are known to possess a wide range of disease preventive properties that are largely attributed to their antioxidant and anti-inflammatory activity (Dykes and Rooney, 2007; Nignpense et al., 2021). Among the major cereal grains, sorghum [*Sorghum bicolor* (L.) Moench] genotypes exhibit an abundant and diverse array of grain polyphenols which are localized in the seed pericarp (external coat) and testa (layer between pericarp and endosperm) (Hahn et al., 1984; Dykes and Rooney, 2007). In black pigmented sorghum grain, 3-deoxyanthocyanidins (3-DOAs) represent the predominant flavonoid (Awika et al., 2004; Dykes et al., 2005, 2009). At present, black sorghum grain represents the only known commercial source of 3-DOAs that are valued for nutraceuticals owing largely to their stability and excellent antioxidant activity (Awika et al., 2004; Xu et al., 2021).

Plant secondary metabolites including polyphenols are widely implicated in contributing to plant immunity playing vital roles as phytoanticipins and stress-induced phytoalexins (Tiku, 2020). Plant defense mechanisms are intricate and specific responses to abiotic and biotic stimuli include the reinforcement of the cell wall, generation of reactive oxygen species (ROS), induction of defense-related proteins, rapid cellular influx of Ca^2+^, production of phytohormones, and the biosynthesis of secondary metabolites (Snyder and Nicholson, 1990; van Loon et al., 2006; Rejeb et al., 2014; Checker et al., 2018; Isah, 2019). The specific profile of secondary metabolites induced by stress is determined by the plant species and the specific stress signal with many studies reporting different compounds synthesized under specific stress conditions (Isah, 2019; Zhuang et al., 2023). In sorghum vegetative tissue, wounding and pathogen stress have been shown to induce the localized accumulation of 3-DOAs at the site of injury (Snyder and Nicholson, 1990; Nielsen et al., 2004; Kawahigashi et al., 2016; Nida et al., 2021). In sorghum genotypes with purple/red secondary plant color (determined by a dominant *P* gene; Kawahigashi et al., 2016), epidermal cells respond to fungal penetration with localized accumulation of 3-DOAs in vesicle-like inclusions directly surrounding the sites of fungal penetration (or mechanical wounding), and 3-DOAs are subsequently released into the apoplastic space where they are proposed to adhere to partially hydrolyzed plant cell wall material (Snyder and Nicholson, 1990; Nielsen et al., 2004).

The environmental conditions that activate 3-DOA biosynthesis in pericarp of black sorghum have been shown to require high-fluence ultraviolet-B (UVB) exposure (Fedenia et al., 2020). Unique from vegetative tissues where 3-DOA biosynthesis is activated by mechanical wounding or pathogen attack, the fluence and duration of pericarp exposure to UVB light determines the extent of 3-DOA accumulation in grain of black sorghum (Fedenia et al., 2020). Unlike the low-fluence UV induction of anthocyanin biosynthesis (Guo et al., 2008; O’Hara et al., 2019), full penetrance of black sorghum pericarp (and the associated activation of 3-DOA biosynthesis) requires extended exposure to UVB fluences normally associated with agricultural field conditions in temperate climates (Pfeiffer and Rooney, 2015; Fedenia et al., 2020). Changes in gene expression during black pericarp development revealed that UVB activates genes related to plant defense and secondary metabolism, suggesting that 3-DOA accumulation is associated with UVB light stress in black pericarp (Fedenia et al., 2020). While UVB is well known as a stress elicitor in many plant species and tissues, the UVB-induced changes in gene expression and 3-DOA biosynthesis were unique to pericarp of black sorghum genotypes as similar UVB-induced changes were not observed in pericarp of red-seeded sorghum (Fedenia et al., 2020). Thus, the black pericarp trait in sorghum represents a case of genetic susceptibility to UV radiation that is not only genotype-specific, but also tissue-specific.

UVB radiation, with wavelength range between 280–315 nm, is recognized as an environmental signal contributing to developmental processes and as a potential stress factor (O’Hara et al., 2019; Shi and Liu, 2021). Plant developmental processes affected by UVB light include hypocotyl elongation, cotyledon expansion, and flavonoid accumulation (Shi and Liu, 2021); however, high fluence UVB can increase ROS levels in the cell that has the potential to damage DNA and lipids, impair photosynthesis, and damage the integrity of the thylakoid membranes (Strid et al., 1994). Plants have developed intricate systems to balance ROS generation and detoxification through enzymatic systems to metabolize cellular ROS (e.g., superoxide dismutase, glutathione S-transferase (GST), and glutathione reductase amongst others) or non-enzymatic detoxification (Saed-Moucheshi et al., 2014; Zhuang et al., 2023). Within the non-enzymatic strategy is the production of protective compounds including flavonoids (Das and Roychoudhury, 2014). In animal cell culture systems, exogenous 3-DOAs (particularly the 7-methoxylated forms of these compounds) have been shown to protect against oxidative stress by scavenging oxidizing molecules and by inducing phase II quinone reductase enzymes (Yang et al., 2009; Awika et al., 2009; González-Montilla et al., 2012). The effect of 3-DOAs in heterologous systems may provide insight for 3-DOAs acting as photoprotective compounds with radical scavenging activity in sorghum pericarp under UV stress (Wu et al., 2023).

Flavonoid biosynthesis is a complex pathway that is comprised of more than 20 structural genes that are regulated by various classes of regulatory genes (Ferreyra et al., 2012). A comprehensive description of the flavonoid pathway leading to 3-DOA biosynthesis has been summarized by Fedenia et al. (2020). By convention, flavonoid biosynthesis has been shown to be transcriptionally regulated by transcription factors (TFs) including Myb proto-oncogene like (MYB), basic Helix-Loop-Helix (bHLH), and WD-40-repeat protein (WD40) along with members of other TF families (Ferreyra et al., 2012; Naik et al., 2022). In sorghum, the *Yellow seed1* (*Y1*) gene encodes an R2R3 MYB TF, which plays a key role in the biosynthesis of 3-DOAs in sorghum vegetative tissue (Ibraheem et al., 2010; Nida et al., 2021). The *Y1* R2R3 MYB TF regulates a suite of structural genes in the flavonoid pathway including chalcone synthase (CHS), chalcone isomerase (CHI), and dihydroflavonol 4-reductase (DFR) (Boddu et al., 2005; Ibraheem et al., 2010). In addition, a WD40 gene in sorghum, *Tannin1*, is involved in the regulation of CHS, CHI, DFR, leucoanthocyanidin reductase and anthocyanidin synthase (ANS) (Wu et al., 2012). As flavonoid biosynthesis is activated by environmental stressors, TFs involved in abiotic and biotic stress signaling pathways have been shown to regulate the transcription of flavonoid biosynthesis structural genes (Ibraheem et al., 2010; Ferreyra et al., 2012). Numerous studies have reviewed the involvement of TF families including MYB, bHLH, WRKY, basic leucine zipper (bZIP), NAM-ATAF1/2-CUC2 (NAC), and AP2/ERF/DREB that regulate plant stress responses through mediating secondary metabolite biosynthesis (Morishita et al., 2009; Ferreyra et al., 2012; Meraj et al., 2020; Mandal et al., 2022). TFs are but one key aspect of the complex interconnected stress signaling pathways that also involve signaling molecules including damage-associated molecular patterns (DAMPs), pathogen-associated molecular patterns (PAMPs), receptor kinases, mitogen-activated protein kinases (MAPKs), plant hormones, and ROS signaling pathways amongst others (Hou et al., 2019; Meraj et al., 2020).

Over the past two decades, gene co-expression networks have been utilized to elucidate gene clusters (modules) whose expression is highly correlated with phenotypes of interest in animal and plant species (for review, see Rao and Dixon, 2019) including secondary metabolic pathways (Mandal et al., 2020). In plant systems, differential gene expression analysis has been routinely utilized to quantify the expression of structural and regulatory genes in response to abiotic and biotic stressors and to examine the tissue- and genotype-specificity of these responses. With the advent of co-expression network ‘omics’ analyses, complex regulatory networks associated with specific physiological responses to biological stress can be better understood. By combining metabolic profiling and high-throughput sequencing of transcriptomes, a better understanding of environmentally regulated flavonoid biosynthesis in pericarp of fruits and cereal grains have been elucidated including those of rice (Yang et al., 2021), maize (Li et al., 2022), barley (Han et al., 2020), sorghum (Zhou et al., 2022), tomato (Sacco et al., 2019), and grape (Li et al., 2023) amongst many others. These studies have provided knowledge of tissue-specific gene networks and gene interconnectivity that include members of flavonoid structural gene families, regulatory genes (TFs), signaling cascades and flavonoid transporters.

To better understand the gene regulatory events associated with 3-DOA biosynthesis in pericarp of black sorghum, a weighted gene co-expression network analysis (WGCNA) was conducted utilizing ultraperformance liquid chromatography (UPLC) quantification of pericarp 3-DOAs in conjunction with RNA-Seq transcriptomics during grain development. In conjunction with the exploratory network analysis, ROS levels in grain of black and red pericarp sorghums were quantified to determine if UV radiation leads to elevated ROS levels and oxidative stress in black pericarp. Finally, we conducted light microscopy studies of developing pericarp tissue to determine if 3-DOAs are associated with the cell wall as a potential reinforcement mechanism to protect the seed and developing embryo from high-fluence UV. Collectively, these analyses provide insight into the genes and gene networks associated with 3-DOA biosynthesis in black pericarp and provide further insight into the high-fluence UV induced gene expression associated with the black pericarp trait in sorghum.

## 2 Materials and methods

### 2.1 Plant material and plant growth conditions

For 3-DOA profiling of pericarp by UPLC and sample preparation for RNA-Seq library construction, sorghum genotypes BTx378 (red pericarp) and RTx3362 (black pericarp) were grown in Conviron BDW Growth rooms (Conviron Products of America, Pembina, ND) under the conditions established and detailed by Fedenia et al. (2020). Briefly, sorghum plants were grown under conditions to reasonably mimic field conditions with photosynthetically active light (VIS) and supplemental UV radiation provided by fluorescent fixtures containing UVA+UVB emitting bulbs (Solacure LLC, Browns Summit, NC). Sorghum panicles were allowed to develop under VIS+UVA+UVB light regime or under cladding materials that filtered out specific regions of the light spectrum. These cladding materials included UV-filtering Plexiglas acrylic sheet OP3/UF-5 (ePlastics^®^, San Diego, CA) to allow exposure to only VIS, Mylar polyester film (U.S. Plastic Corp.^®^, Lima, OH) to allow both VIS and UVA light, and acrylic-latex foam-coated “blackout” fabric (Tri-Tex Enterprises, Inc., Dallas, TX) to block all light exposure of the panicles. At specific time points during grain development, pericarp tissue was mechanically excised with razor blades and immediately placed in liquid nitrogen (LN_2_) prior to RNA isolation or sample preparation for 3-DOA profiling. This experimental trial was repeated in time (although BTx378 was excluded from the second trial) under the same growth conditions resulting in a total of 50 unique genotype/light spectrum combinations for pericarp RNA-Seq analysis and 3-DOA quantification.

To quantify grain ROS levels and conduct a light microscopy study of developing grain, genotypes RTx3362 and BTx378 were planted at the Texas A&M AgriLife Research farm in College Station, TX (latitude and longitude coordinates; 30.55, -96.44) in April 2023 and grown using standard agronomic practices for grain sorghum. At full anthesis, panicles were either covered with a pollination bag (MIDCO Global, St. Louis, MO) to block exposure to UV light, or panicles were allowed to develop under full sunlight. RTx3362 and BTx378 developing grains were sampled at 5, 10, and 17 days after anthesis (DAA) with grain collected on clear days in late June and early July (between 9-10:30 am). For ROS determinations (see protocol below), individual grains were removed from the panicle, glooms discarded, and grain immediately placed in LN_2_ in the field prior to transport to storage at -80°C until sample processing. For light microscopy studies, grains were hand harvested and were placed on wet ice prior to transport to the laboratory for tissue fixation (see protocol below).

### 2.2 ROS assays

To quantify ROS levels of field-grown sorghum grain, a dichlorofluorescein ROS/RNS Assay Kit (Abcam Inc., Boston, MA) was utilized according to the manufacturer’s protocol. In brief, flash frozen (in LN_2_) grain of genotypes BTx378 and RTx3362 (15 grain per each ROS assay) from each panicle were ground to a fine powder in LN_2_ and then added to phosphate-buffered saline buffer (15 mg ground tissue/mL buffer). Samples were vortexed (30 sec), centrifuged (10,000g, 10 min at 4°C), and 250 µl of clarified supernatant was subsequently serial diluted (1:1, four times with phosphate-buffered saline buffer) to a final 16-fold dilution. Fifty µl aliquots of a diluted sample were added to four wells of a black 96-well plate (Greiner Bio-One International GmbH, Monroe, NC) and then 50 µl of catalyst plus 100 µl of fluorescent dye (or 100 µl water for blanks) were added to each sample well. Each plate assay represented an experimental replicate containing a complete factorial of genotype (BTx378, RTx3362), light (full sunlight, shaded with a pollination bag), and timepoint during grain development (5, 10, 17 DAA). Two technical replicates of genotype/light treatment/DAA combinations were added to each plate while 3 biological replications (three plate assays) were conducted. In addition, a serial dilution of H_2_O_2_ was included in each plate for calculating a standard curve. Fluorescence was determined after a 45-min incubation period in the dark on a Molecular Devices SpectraMaxi D3 multiplate reader (Molecular Devices, LLC., San Jose, CA) with excitation at 480 nm and emission at 530 nm with the automatic setting and 2 nm laser distance.

For ROS data analysis, readings from each plate were treated as an experimental replicate, and a standard curve based on the serial dilution of H_2_O_2_ was calculated for each plate. Technical replicates for each genotype/light treatment/DAA combination were averaged to represent an observation utilizing the H_2_O_2_ standard curve, and fluorescence readings were converted to μmol H_2_O_2_·mg FW^-1^ (fresh weight) and reported as the average of three biological replications ( $\bar{\text{X}}$ ± std error). A two-way ANOVA was performed in R (v4.2.1) using the lme4 package (v1.1-34) (Bates et al., 2015) on log transformed values followed by Tukey’s HSD to determine differences in ROS levels in sorghum caryopses. The main effects of DAA, light treatment and genotype were considered fixed while replication was considered random (y = light treatment + DAA + genotype + (DAA x light treatment) + (genotype x light treatment) + (genotype x DAA) + error).

### 2.3 Sample preparation for light microscopy

At specific stages of grain maturation, grain from the sorghum genotypes BTx378 and RTx3362 were sampled from field grown plants under either full sunlight or under pollination bags to block UV light. Grains were fixed in formaldehyde alcohol acetic acid (FAA) (3.7% deionized formaldehyde, 50% ethanol, 5% glacial acetic acid) by vacuum infiltration and then placed overnight in fresh FAA at 4°C. Following fixation, pericarp tissues were mechanically excised with a razor blade and placed in fresh FAA. Excised pericarp sections were then dehydrated in a concentration series of ethanol followed by a tertiary butyl alcohol concentration series and finally in a mixture of paraffin oil in tertiary butyl alcohol (1:1). The dehydrated pericarp tissues were embedded in TissuePrep embedding media (Thermo Fisher Scientific, Waltham, MA) and sectioned on a LKB Bromma 2218 HistoRange rotary microtome (section thickness 20 µm). Section ribbons were adhered to glass slides dipped in Surgipath Sta-on (Leica Biosystems, Wetzlar, DE), heated to 50°C overnight and then stored at room temperature prior to dewaxing and staining.

For visualizing flavonoids, sorghum pericarp tissue sections were dewaxed in xylene, partially rehydrated in ethanol (100% followed by 50%), and flavonoids stained with Diphenylborinic acid 2-aminoethyl ester (DPBA) (1% DPBA solution in methanol). Pericarp sections were incubated in DPBA (1 min) followed by adding a drop of distilled water to prevent sections from drying. Fluorescence was visualized upon excitation with UV light (ex. 365 nm band pass, em. 400 nm long pass) using a Zeiss Axiophot microscope (Zeiss, Oberkochen, Baden-Wurttemberg, Germany) equipped with a custom DAPI longpass filter set (Chroma Technology Corp., Bellows Falls, VT).

### 2.4 Transcriptome sequencing and analysis

Total RNA from pericarp samples was isolated, quantified and RNA integrity accessed as detailed by Fedenia et al. (2020). Illumina template library preparation using the TruSeq Stranded Total RNA library kit (Illumina Inc., San Diego, CA) and sequencing (paired-end; 2×150 bp) were performed for 6 biological replications per treatment of the black-pericarp genotype RTx3362 and 2 biological replications for the treatment of the red-pericarp genotype BTx378 on a NovaSeq 6000 (Illumina, Inc., San Diego, CA). Sequence cluster identification, quality pre-filtering, base calling, and uncertainty assessment were conducted in real time using Illumina’s HCS 2.2.58 and RTA 1.18.64 software and the resulting reads were analyzed for quality and quantity using FastQC Software (v0.11.5) (Andrews, 2010). Trimmomatic (Bolger et al., 2014) was used for the removal of primers, adapters, and other contaminate sequences from the fastq files using the following command line parameters: ILLUMINACLIP: TruSeq3 PE.fa:2:20:10:4:TRUE SLIDINGWINDOW:4:15 HEADCROP:10 TRAILING:3 MINLEN:36. Trimmed fastq files for each RNA-Seq library were imported into the CLC Genomics Workbench (v22.0.2) (Qiagen, Valencia, CA) and mapped to the *Sorghum bicolor* v5.1 reference genome (https://phytozome-next.jgi.doe.gov/_info_/Sbicolor_v5_1) with the RNA-Seq tool. The RNA-Seq Analysis tool was used for stranded libraries to produce gene expression and transcript expression track files with the following parameters:

Reference sequence Sbicolor_730_v5.0 (Genome); Gene Track Sbicolor_730_v5.0 (Gene); mRNA track Sbicolor_730_v5.0 (mRNA); Use spike-in controls = No; Mismatch cost = 2; Insertion cost = 3; Deletion cost = 3; Length fraction = 0.8; Similarity fraction = 0.8; Global alignment = No; Strand specific = reverse; Library type = Bulk; Maximum number of hits for a read = 10; Count paired ends as two = No; Ignore broken pairs = Yes; Expression value = TPM; Calculate expression for genes without transcripts = No; Create reads track = Yes; Create report = Yes; Create fusion gene table = No; Create list of unmapped reads = No.

Differential gene expression was also performed in the CLC Genomics Workbench with the Differential Expression for RNA-Seq 2.7 Analysis tool between RTx3362 and BTx378 under VIS+UVA+UVB at the four time points with the following parameters:

Technology = Whole transcriptome; RNA-Seq filter on average expression for false discovery rate (FDR) correction = Yes; Test differential gene expression due to = Genotype while controlling for = Biological rep; Sequencer/Library Prep Comparisons = against control group; Control group = BTx378.

### 2.5 3-DOA quantification by UPLC with photodiode array detection

For 3-DOA quantification, excised pericarp tissues were ground with a mortar and pestle under LN_2_, frozen tissues lyophilized, and 3-DOAs were extracted and quantified as reported previously by Fedenia et al. (2020). Briefly, sorghum 3-DOAs were identified based on the retention times of commercial standards and UV-Vis spectra. The commercial standards that included luteolinidin chloride, apigeninidin chloride, and 7-methoxyapigeninidin chloride, were obtained from ChromaDex (Irvine, CA). Sorghum 3-DOAs were quantified as described by Dykes et al. (2009, 2013). Extracts were analyzed on a Nexera-i LC-2040C 3D liquid chromatograph equipped with a photodiode array detector (Shimadzu Scientific Instruments, Kyoto, Japan) with 3-DOAs separated using a Kinetex C18 column (150 × 2.1 mm, 2.6) (Phenomenex, Torrance, CA). The 3-DOA concentrations (μg/g dry weight) were expressed as means of two technical replications per each biological replicate.

### 2.6 Statistical analyses

Using 3-DOA concentrations from two replicated experiments combined, a two-way analysis of variance (ANOVA) was run in R (v4.2.1) using the lme4 package (v1.1-34) (Bates et al., 2015). Log transformed values for luteolinidin (LUT), apigeninidin (API), 5-methoxy-luteolinidin (5-MeLu), 7-methoxy-apigeninidin (7-MeAp), and total 3-DOA concentrations were used to determine the effect of experimental runs and replications for downstream utilization in gene expression network construction. All models were considered fixed effect containing the main effects of experiment and replication: Model (A) RTx3362 and BTx378 pericarp under VIS+UVA+UVB light at 10 DAA and 17 DAA (y = experiment + DAA + genotype + replication + (DAA x genotype) + (experiment x DAA) + error); model (B) RTx3362 under VIS+UVA+UVB at 10 DAA and 17 DAA (y = experiment + DAA + light treatment + replication + (DAA x light treatment) + (experiment x DAA) + (experiment x light treatment) + error); model (C) RTx3362 pericarp exposed to each light treatment (VIS, VIS+UVA, VIS+UVA+UVB) at 17 DAA (y = experiment + light treatment + replication + (experiment x light treatment) + error); model (D) RTx3362 and BTx378 pericarp exposed to VIS+UVA+UVB light at 17 DAA (y = experiment + genotype + replication + error).

### 2.7 Network construction and *Cis* regulatory motif analysis

To correct batch effects between sequencing experiments in the combined analysis, empirical Bayes moderated linear model adjustment was used through the function in the WGCNA package (Langfelder and Horvath, 2008). Genes that had more than one transcript per kilobase million (TPM) in at least 3 of the 50 samples were considered for the network analysis totaling 21,532 genes. The co-expression gene network was constructed from the filtered and corrected gene expression data using the WGCNA R package (v1.72.1) (Langfelder and Horvath, 2008, 2012) in R (v4.3.0). A matrix was constructed of pairwise comparisons using Pearson’s correlation coefficient (r) between the genes and transformed into an adjacency matrix using the “signed” method. A soft threshold of 20 was chosen based on the scale-free topology index threshold (R^2^ > 0.89) and low mean connectivity. To detect modules of genes with similar expression profiles, genes were clustered into a dendrogram using hierarchical clustering from the weighted matrix. A module eigengene was defined to represent the gene expression profile of the module and the correlation of that gene to its module was calculated as a module membership (MM). The eigengenes were then correlated to 3-DOA concentrations (μg/g dry weight) of each sample and the mean expression values (TPM) of 6 (bait) genes with roles in the 3-DOA biosynthetic pathway. The gene significance (GS) for each gene within its module was calculated as the correlation between gene expression and the 3-DOA concentrations or with bait gene expression. The hub genes were selected based on their intramodular connectivity (> 0.20).

DNA-binding motif analysis was performed on the promoter regions of the Darkgrey and Saddlebrown WGCNA modules by extracting 2 kilobase upstream DNA sequence from the transcriptional start site and running them through the MEME suite (Bailey et al., 2015) with the following parameters:

meme -objfun de -test mhg -dna -revcomp -mod anr -brief 100000 -p 16 -evt 0.01 -nmotifs 30 -minw [6,7,8,10] -maxw 50 -neg promoter_control_file

Where the `-minw [6,7,8,10]` parameter indicates that 4 separate runs were performed with the -minw value set to either 6, 7, 8, or 10. The `-neg promoter_control_file` is a fasta file that has the 2 kilobase upstream sequences from the transcriptional start site for all annotated genes in the BTx623 v5.1 genome; sequences were extracted using the GrameneMart feature from the Gramene database (Tello-Ruiz et al., 2022). Each enriched DNA-binding motif was compared via the TomTom program (Gupta et al., 2007) against the JASPAR non-redundant plant database (Fornes et al., 2020) to assess similarity to previously characterized DNA-binding sequences. The position weight matrices from the MEME-produced analysis in the top-correlated genes of the Darkgrey module were then plugged into the FIMO analysis (Grant et al., 2011) to reciprocally map to any motif occurrences that did not come out in the initial MEME analysis. Gene Ontology (GO) enrichment analysis was performed using the GO tool from PlantTFDB 4.0 (Jin et al., 2017) according to the *Sorghum bicolor* annotation of genotype BTx623 (Sbicolor_454 v3.0.1; www.phytozome.jgi.doe.gov).

## 3 Results

### 3.1 ROS determination in pericarp tissue

**Figure 1:**
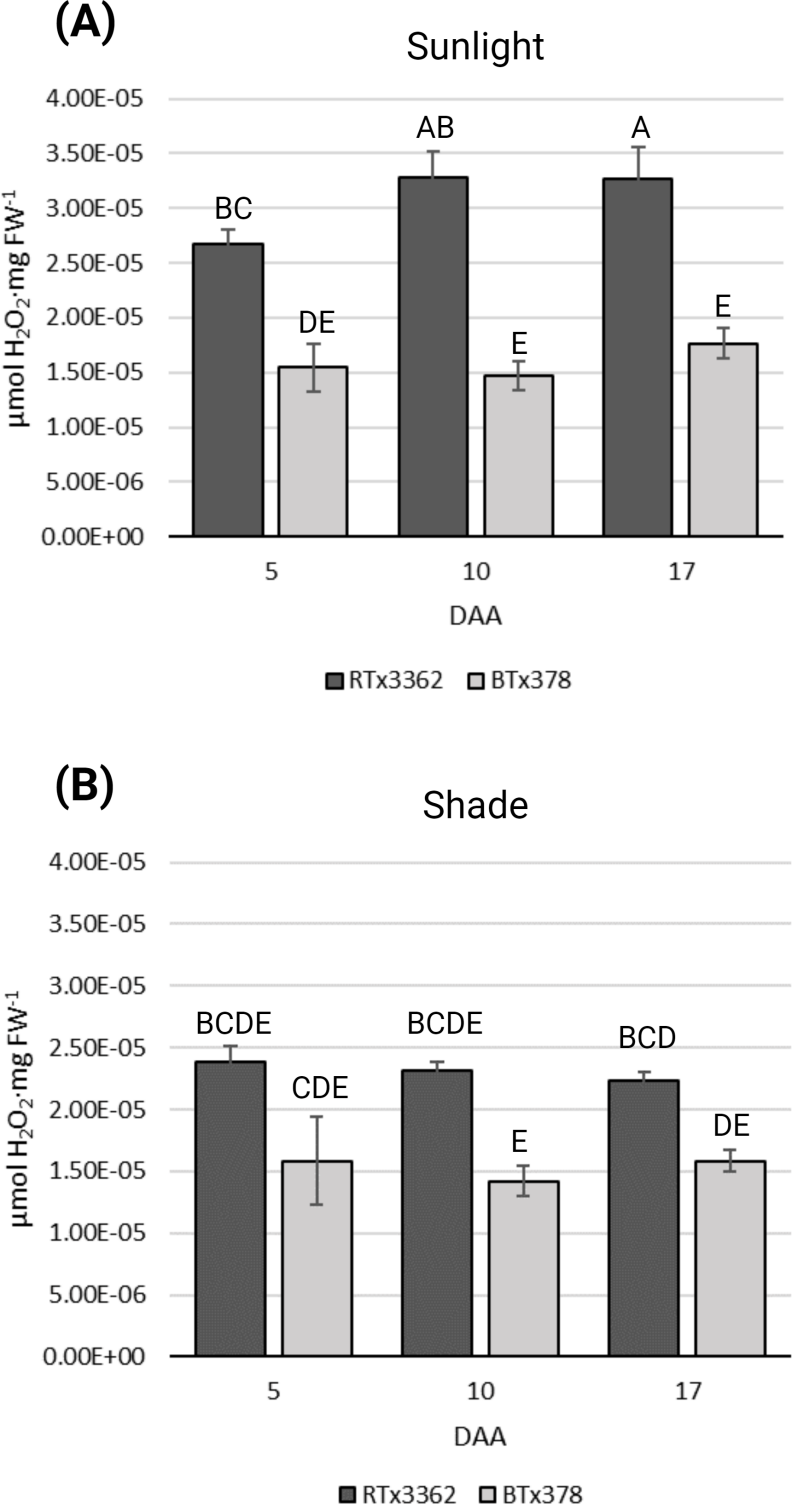
ROS levels during grain maturation of black and red pericarp sorghum. ROS determinations were measured as H_2_O_2_ equivalents (μmol H_2_O_2_·mg FW^-1^) from three biological replications of grain from black (RTx3362) and red (BTx378) sorghum genotypes sampled 5, 10, and 17 DAA. **(A)** Panicle matured in sunlight and **(B)** panicle matured under a pollination bag. Levels not connected by the same letters are significantly different (*p* < 0.05) based on Tukey’s HSD.

To determine if the genotype-specific accumulation of 3-DOAs in pericarp of black sorghum is associated with elevated cellular ROS levels, grain of field-grown black (RTx3362) and red (BTx378) pericarp genotypes were examined during grain development under full sunlight or under shade conditions that blocked UV radiation. ROS levels in black sorghum grain were higher than for red seeded sorghum under full sunlight at 5, 10, and 17 DAA (**Figure 1A**). ANOVA revealed that sorghum grain showed genotypic differences (*p* < 0.05) that were dependent on the light condition (**Supplementary Table 1**). In black sorghum, ROS levels increased from 5 to 10 DAA and remained high at 17 DAA under full sunlight. Grain of the red seeded sorghum genotype did not show significant differences in ROS levels under full sunlight (**Figure 1A**) or shaded conditions (**Figure 1B**) at the three sampling dates. These results show that black sorghum exhibits higher cellular ROS levels than red sorghum under full sunlight during early grain development that coincided with the appearance of the black pigmentation in grain beginning at 10 DAA (Fedenia et al., 2020).

### 3.2 Fluorescent microscopy of sorghum pericarp

**Figure 2:**
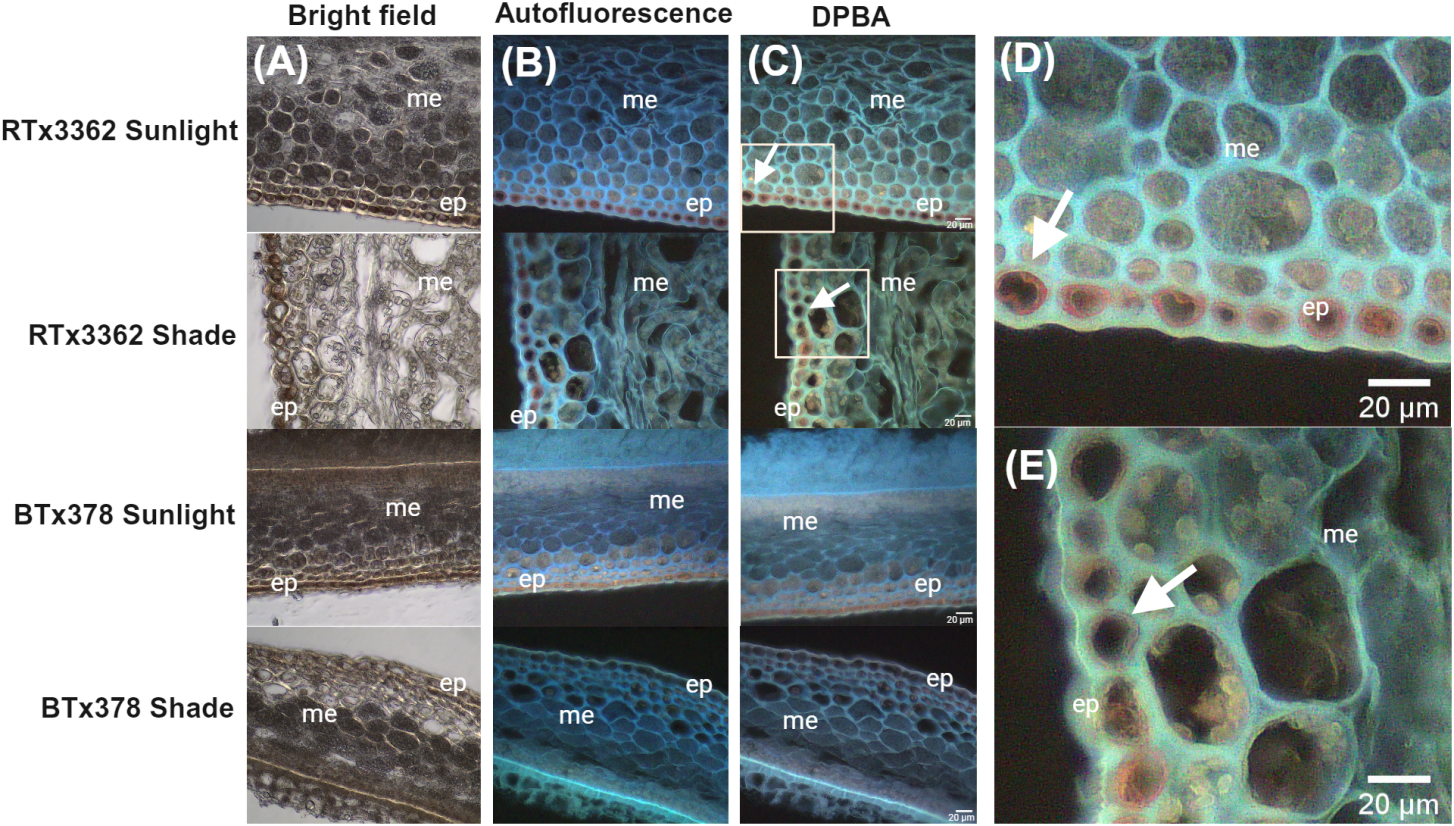
Light micrographs of pericarp from red and black grain sorghum. Micrographs of pericarp developed under sunlight or shaded at 11 DAA. **(A)** Bright field micrographs of unstained cross sections of pericarp showing the epicarp (ep) and mesocarp (me) photographed under white light. **(B)** Autofluorescence micrographs of pericarp (unstained) photographed under UV excitation with cell walls visualized as blue autofluorescence. **(C)** Micrograph of flavonoid yellow-green fluorescence of DPBA-treated sections with white arrows marking the association of flavonoid fluorescence with epicarp cell layer in RTx3362 pericarp. **(D)** and **(E)** higher magnification with the area visible marked by the white rectangle of RTx3362 pericarp in panel **(C)**. White arrows in **(D, E)** mark DPBA-stained flavonoids localizing near the cell wall in the epidermal layer of the RTx3362 pericarp. Scale bar = 20 µm.

To determine where 3-DOAs are localized in the pericarp of black sorghum, we conducted a light microscopy study of fixed pericarp of black and red sorghum at 11 DAA under full sunlight or shaded conditions (**Figure 2**). Bright field (**Figure 2A**), autofluorescent (**Figure 2B**) and DPBA stained (**Figure 2C**) images of cross-sectioned pericarp tissue visualized through fluorescent light microscopy showed flavonoid accumulation especially in the epicarp of the grain as revealed by DPBA staining (**Figure 2C**). In black pericarp genotype RTx3362, imaging revealed that the cells of the epicarp accumulated visible flavonoid pigments under sunlight and shaded conditions, which is consistent with the reported detection of 3-DOAs in pericarp of RTx3362 at this stage of grain development (Fedenia et al., 2020). Flavonoids stained with DPBA developed a green hue (**Figure 2C**) that colocalized with the autofluorescent lignin within cell walls (**Figure 2B**). Under higher magnification (**Figures 2D, E**) DPBA-stained flavonoids in the epidermal layer became a bright orange that appears to be co-localized with the cell wall of the epicarp, specifically the epidermal cells. By comparison, in red grain genotype BTx378, DPBA fluorescent staining of flavonoids could be seen in the tissue exposed to sunlight although the fluorescent signal was far less evident, especially in the shaded tissue where the DPBA fluorescent signal was nearly undetectable (**Figure 2C**). The lack of DPBA fluorescent signal in shaded BTx378 pericarp agrees with the observation that BTx378 grain is largely devoid of red pigmentation under blackout lining indicating flavonoid biosynthesis is suppressed in shaded conditions. Therefore, the present light microscopy study suggests that a detectable fraction of the flavonoids in black sorghum pericarp are co-localized with the cell wall and the flavonoid signal was far more prevalent in black vs. red pericarp epidermal cells.

### 3.3 Differential gene expression during sorghum grain development

The present study examined a time series during grain development commencing prior to the accumulation of detectable levels of 3-DOAs in pericarp tissues (2 and 5 DAA) and extended to a developmental stage where pericarp 3-DOA accumulation has plateaued (17 DAA). RNA-Seq libraries from pericarp tissue of RTx3362 and BTx378 under VIS+UVA+UVB light treatments at 2, 5, 10, and 17 DAA were compared through differential gene expression analysis. Across the 4 sampling times, pairwise comparisons between the two genotypes detected a total of 26,640 differentially expressed genes (DEGs) (FDR *p*< 0.05, fold change cutoff of log_2_> 1 for upregulated genes or < -1 for downregulated genes) with 6,344 genes differentially expressed at 2 DAA, 5,053 at 5 DAA, 6,767 at 10 DAA, and 8,476 at 17 DAA (**Supplementary Data Sheet 1**).

**Figure 3:**
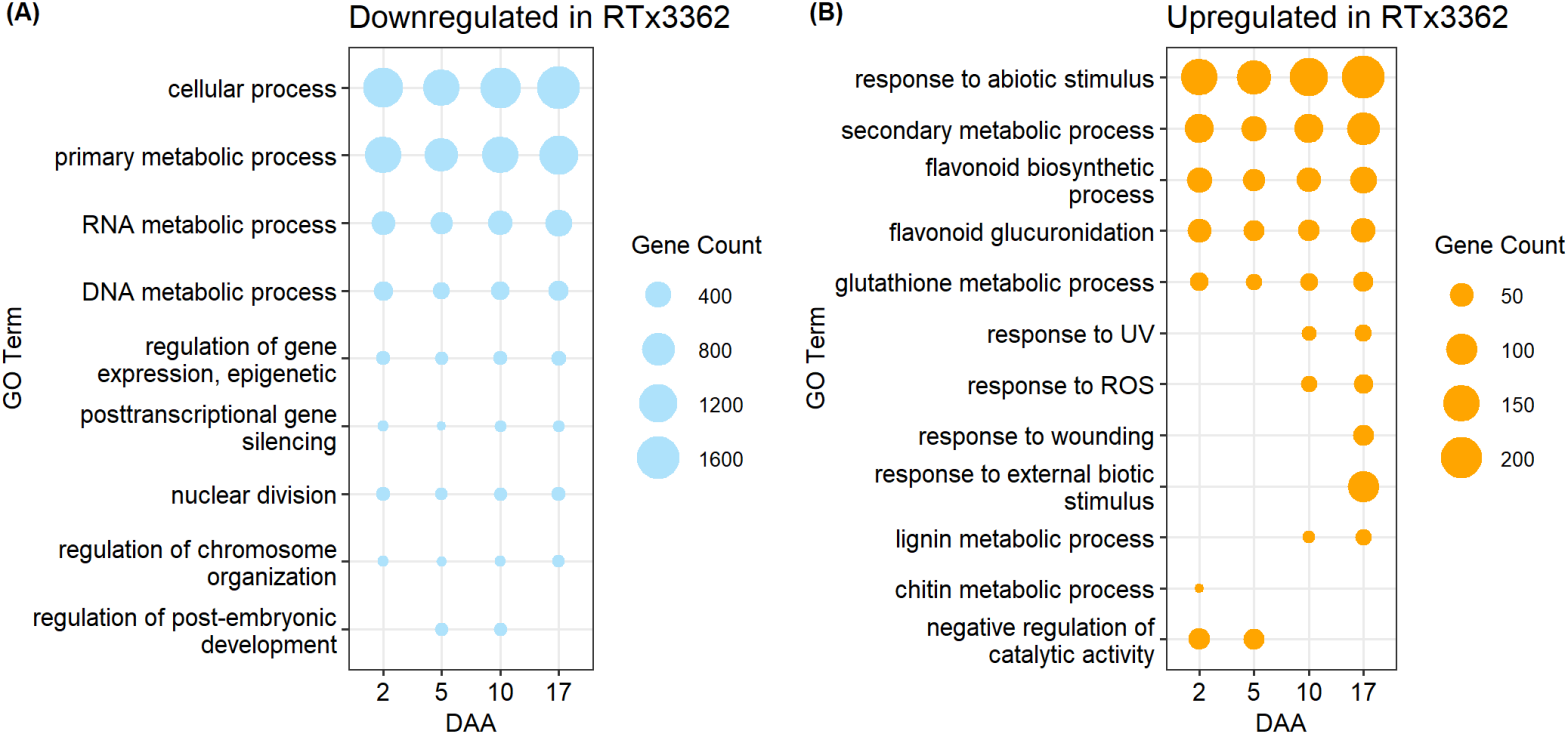
GO terms for biological processes enriched in **(A)** downregulated or **(B)** upregulated DEGs in pericarp of black sorghum. A subset of significant GO terms is shown while a complete list of GO terms, GO IDs, and associated *p*-values are given in **Supplementary Data Sheet 2**. Grain developed under a light spectrum of VIS+UVA+UVB and pericarp samples were harvested 2, 5, 10, and 17 DAA. Upregulated or downregulated refers to gene expression in genotype RTx3362 with genotype BTx378 as the reference.

GO terms for biological processes (P) comparing expression during pericarp development in black vs. red genotypes revealed marked differences between the two pericarp types. An abbreviated list of GO terms is shown in **Figure 3** while the complete list of significant GO terms is given in **Supplementary Data Sheet 2**. Under a light spectrum including VIS+UVA+UVB, the GO categories of cellular process (GO:0009987) and primary metabolic process (GO:0044238) were downregulated in black pericarp vs. red pericarp across the 4 developmental stages (**Figure 3A**). In addition, GO categories associated with RNA and DNA metabolic processes (GO:0016070, GO:0006259), and regulation of gene expression (GO:0040029, GO:0016441) were downregulated in black pericarp, amongst others. Finally, additional downregulated genes were associated with the GO categories of nuclear division (GO:0000280), regulation of chromosome organization (GO:0033044), and regulation of post-embryonic development (GO:0048580). By contrast, GO categories upregulated in black pericarp tissues across all timepoints included response to abiotic stress stimulus (GO:0009628), secondary metabolic process (GO:0019748) and glutathione metabolic process (GO:0006749) (**Figure 3B**). As anticipated, flavonoid metabolic processes were upregulated across the 4 timepoints including flavonoid biosynthesis (GO:0009813) and flavonoid glucuronidation (GO:0052696). In black pericarp tissues, a series of GO categories were associated with genes upregulated at later stages of pericarp development (10 or 17 DAA) that included response to UV (GO:0009411), response to ROS (GO:0000302), response to wounding (GO:0009611), response to external biotic stimulus (GO:0043207), and genes associated with lignin metabolism (GO:0009808). Finally, amongst the genes transiently upregulated early during pericarp development (2 or 5 DAA) were chitin metabolic process (GO:0006030) and genes associated with the negative regulation of catalytic activity (GO:0043086). While a very limited number of the significant GO categories were mentioned herein, this list and the full array of GO categories (**Supplementary Data Sheet 2**) provided evidence that the cellular metabolism in black pericarp tissue is responding to the stress imposed by UV light.

### 3.4 WGCNA of pericarp 3-DOA levels

A WGCNA was used to identify those co-expressed genes whose expression is correlated with 3-DOA levels in pericarp tissue, and to identify hub genes that showed the most connections within modules highly correlated with pericarp 3-DOA levels. In addition to utilizing mean 3-DOA concentrations in network construction, 6 genes that are reported to be involved in flavonoid biosynthesis in sorghum were included as bait genes for the WGCNA including DFR genes Sobic.009G043800 and Sobic.004G050200, CHS Sobic.005G136300, CHI Sobic.001G035600, and flavonoid 3'- hydroxylase/monooxygenase (F3'H) genes Sobic.004G200900 and Sobic.004G200744 (**Supplementary Data Sheet 3**).

ANOVA results on 3-DOA concentrations showed significant differences (*p* < 0.05) in 3-DOA concentrations between genotypes, light treatments, as well as sampling dates (DAA) dependent on genotype and light treatment (**Supplementary Data Sheet 4**). Differences between the two experiments for 3-DOA concentrations were found in all models as well as an interaction between experiment and sampling time in model A and B and experiment and light treatment in model C (**Supplementary Data Sheet 4**). Due to these differences in 3-DOA concentrations between experiments, analyses were constructed for each experiment in addition to a combined meta-analysis across the two experiments.

**Figure 4:**
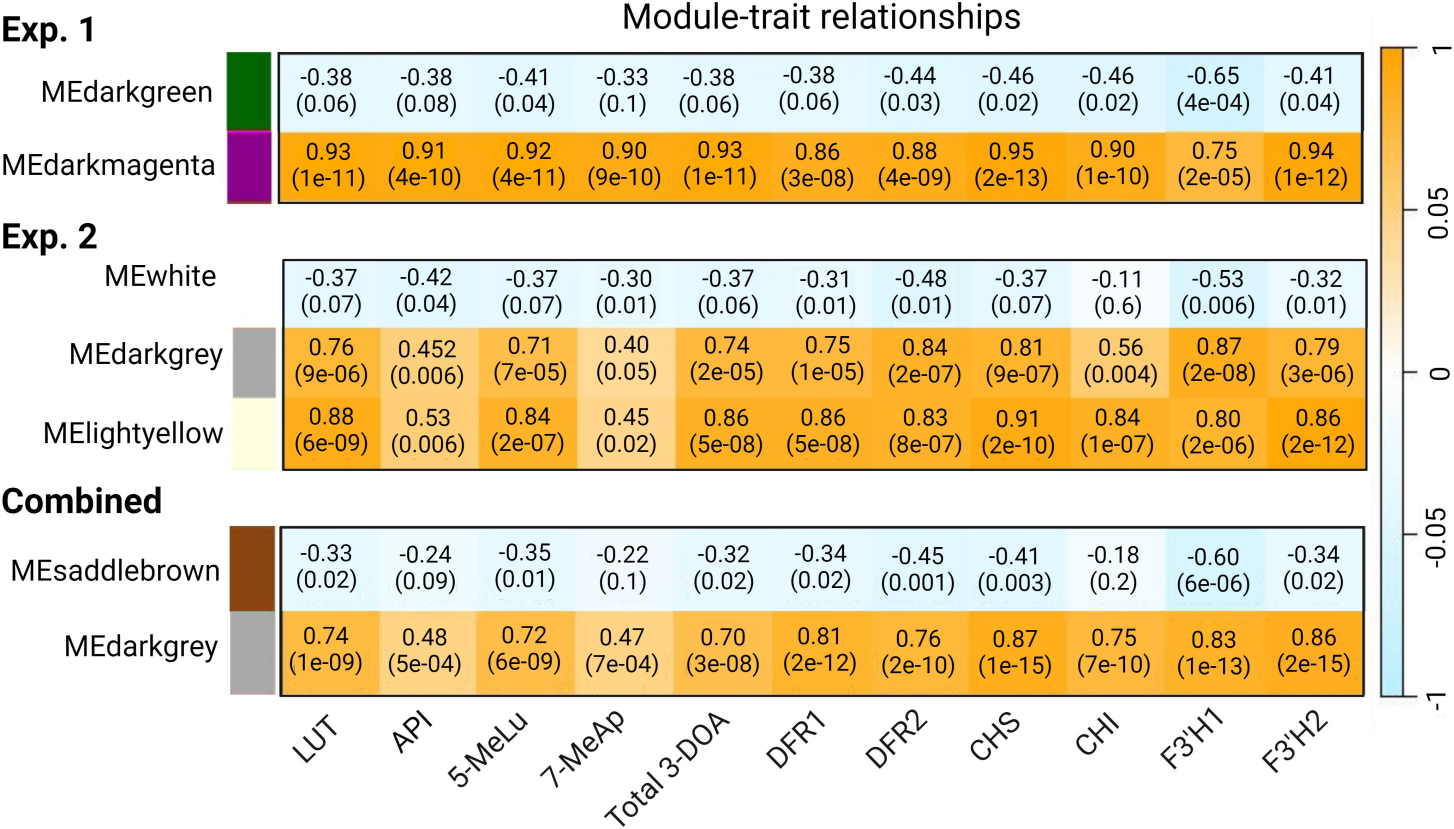
WGCNA module-trait relationships between gene modules and sorghum pericarp 3-DOA levels as well as the expression profiles of six selected bait genes in the 3-DOA biosynthetic pathway. Module-trait relationships are shown for WGCNA for experiment 1 (exp. 1), experiment 2 (exp. 2), and the combined analysis. Through WGCNA a module eigengene that is representative of the expression profile for each module (first principal component) was correlated using Pearson’s correlation coefficient (r) to the expression profiles (TPM) of the six flavonoid biosynthetic bait genes and 3-DOA concentrations in pericarp tissue; *p*-values are shown in parentheses.

Using pericarp 3-DOA levels as the phenotype, 4 WGCNA modules with strong correlation to LUT ranging from r=0.74 (*p*=1e^-09^) to 0.93 (*p*=1e^-11^) were found across each replicated study and the combined network analysis (**Figure 4**). Our initial examination of WGCNA was focused on gene models whose expression was highly correlated with total pericarp 3-DOA levels, and a subset of gene models (r≥0.85) from the combined WGCNA analysis can be found in **Supplementary Data Sheet 5**. With several notable exceptions, the genes highly correlated with pericarp 3-DOA levels were consistent across each replicated study and the combined network analysis as revealed by the GS of total 3-DOAs of 35 of the most highly correlated genes (**Supplementary Data Sheet 5**). The results from the first experiment revealed 45 genes correlated at r≥0.90 with total 3-DOA levels while the second experiment revealed 39 genes correlated at r≥0.90 (**Supplementary Data Sheets 6, 7**). The combined statistical network analysis revealed fewer genes (24 genes) correlated at r≥0.90 with total 3-DOA levels in pericarp likely due to variations associated with plant growth and/or with variations in the chamber conditions between the two replicated studies (**Supplementary Data Sheet 5**). Nevertheless, many of the same gene models were highly correlated with pericarp 3-DOA levels across all three statistical network analyses (**Supplementary Data Sheets 5–7**). Based on this, subsequent results were focused solely on the combined meta WGCNA analysis.

Fifteen WGCNA modules were identified from the combined analysis, with the Darkgrey module having the highest correlation with each of the four 3-DOAs (LUT, API, 5-MeLu, 7-MeAp) and to the total 3-DOA content of sorghum pericarp (**Figure 4**). The correlations ranged from a low of r=0.47 (*p*=7e^-04^) for 7-MeAp to a high of r=0.74 (*p*=1e^-09^) for LUT. In addition, the Darkgrey module which contained 526 genes, showed the strongest association (r=0.75 to 0.87) when the network analysis was conducted using the 6 flavonoid biosynthesis structural genes as bait genes. Among the modules identified from the combined analysis, 5 clusters contained less than 500 genes, 3 with 500-1000 genes, and 7 had greater than 1000 genes. GO enrichment analysis was performed on all WGCNA modules identified from the combined analysis to gain functional insight into the modules (**Supplementary Data Sheet 8**). GO enrichment of the Darkgrey module revealed 42 enriched terms for biological process. Among the top 10 GO categories with the most gene counts were secondary metabolic process (GO:0019748), defense response (GO:0006952), oxidation-reduction process (GO:0055114) and response to biotic stimulus (GO:0009607). Twenty-nine terms for molecular function were found in the module with catalytic activity (GO:0003824), oxidoreductase activity (GO:0016491), and ATP binding (GO:0005524) having the most genes, followed by iron ion binding (GO:0005506) which included genes encoding a series of iron-containing enzymes such as Cytochrome P450s and F3’Hs, amongst others (**Supplementary Data Sheet 8**).

### 3.5 Pathways and specific genes associated with UVB-induced 3-DOA biosynthesis in black pericarp

#### 3.5.1 Flavonoid biosynthesis, secondary metabolism, and metabolic transporter genes

**Figure 5:**
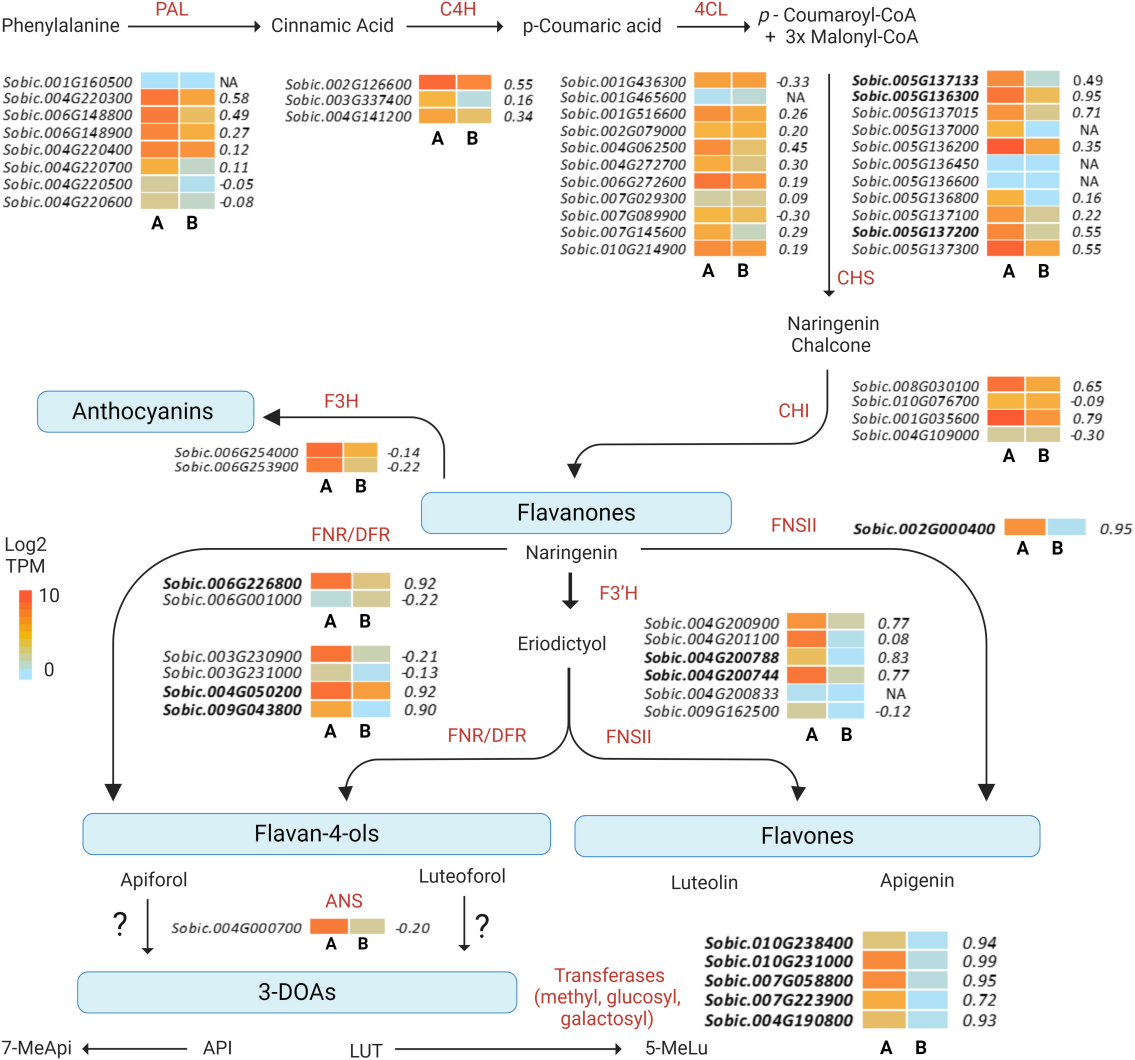
Heat map of differentially expressed genes in the sorghum flavonoid biosynthetic pathway. Heat map of gene expression (TPM) profiles of the gene models for each enzymatic step are represented as the means of biological replicates in **(A)** black (RTx3362) and **(B)** red (BTx378) pericarp after 17 days of exposure to VIS+UVA+UVB light. Gene expression correlation significance with pericarp LUT levels (GS.LUT) are shown to the right of heat maps for each gene model. Hub gene models for the Darkgrey module are shown in bold text. NA refers to genes not included in the analysis due to failure to meet established thresholds for inclusion in the analysis (e.g., low expression, minimum sample number required).

The combined WGCNA permitted the identification of 83 hub genes from the Darkgrey module based on their intramodular connectivity and the correlation of gene expression with pericarp LUT levels. A representative subset of 45 hub genes is presented in **Table 1** while the exhaustive list can be found in **Supplementary Table 2**. In addition, the WGCNA allowed for identification of specific early and late flavonoid biosynthetic pathway family members whose expression are correlated with 3-DOA accumulation in pericarp of sorghum (**Figure 5**) while also identifying specific biosynthetic gene models that represented highly connected hub genes.

**Table 1:** Representative subset of hub genes in the Darkgrey WGCNA module from the combined analysis displaying correlations to pericarp LUT levels (G.S.LUT) and the number of gene connections at weight threshold > 0.2.

Gene Name	Gene Description^a^	Function^b^	G.S.LUT	Connections
**Sobic.005G136300**	CHS WHP1	Flavonoid biosynthesis	0.95	50
**Sobic.005G137133**	CHS	Flavonoid biosynthesis	0.49	10
**Sobic.005G137200**	CHS WHP1	Flavonoid biosynthesis	0.55	14
**Sobic.002G000400**	FNSII	Flavonoid biosynthesis	0.95	49
**Sobic.006G226800**	FNR, *P* gene	Flavonoid biosynthesis	0.92	32
**Sobic.004G200744**	F3’H	Flavonoid biosynthesis	0.77	33
**Sobic.004G200788**	F3’H	Flavonoid biosynthesis	0.83	15
**Sobic.009G043800**	DFR	Flavonoid biosynthesis	0.90	21
**Sobic.004G050200**	DFR	Flavonoid biosynthesis	0.92	10
**Sobic.004G190800**	Galactosyltransferase	Secondary metabolism	0.93	42
**Sobic.007G058800**	*O*-Methyltransferase	Secondary metabolism	0.95	31
**Sobic.010G231000**	*O*-Methyltransferase	Secondary metabolism	0.99	42
**Sobic.010G238400**	*O*-glucosyltransferase	Secondary metabolism	0.94	23
**Sobic.007G223900**	UDP-glucosyl transferase	Secondary metabolism	0.72	29
**Sobic.007G141200**	Cinnamoyl-CoA reductase	Secondary metabolism	0.94	41
**Sobic.001G071000**	NAC Domain	TF	0.95	45
**Sobic.008G183900**	NAC Domain	TF	0.88	29
**Sobic.003G031100**	bHLH	TF	0.97	41
**Sobic.010G035300**	WRKY72	TF	0.90	36
**Sobic.003G349600**	C2H2-type zinc finger	TF	0.88	35
**Sobic.009G164600**	C2H2-type zinc finger	TF	0.60	18
**Sobic.009G234100**	WRKY	TF	0.73	1
**Sobic.005G212700**	GST	Metabolic transport	0.85	19
**Sobic.003G216232**	ABC transporter	Metabolic transport	0.72	19
**Sobic.003G216000**	ABC transporter	Metabolic transport	0.93	39
**Sobic.009G012900**	LRR	Cellular signaling	0.97	43
**Sobic.001G074800**	Receptor-type protein kinase LRK1	Cellular signaling	0.86	22
**Sobic.005G126200**	Brassinosteroid-insensitive associated receptor kinase glycoprotein precursor	Cellular signaling	0.87	19
**Sobic.002G024100**	Lectin-domain receptor-like kinase	Cellular signaling	0.84	18
**Sobic.002G024300**	Lectin-domain receptor-like kinase	Cellular signaling	0.85	15
**Sobic.006G208300**	Auxin induced like protein	Cellular signaling	0.85	12
**Sobic.006G229600**	Lectin-domain receptor-like kinase	Cellular signaling	0.78	2
**Sobic.001G401300**	Pathogenesis-related protein 10a	Stress defense response	0.88	56
**Sobic.005G101600**	Nucleoporin-related//Dirigent protein	Stress defense response	0.89	45
**Sobic.005G101800**	Nucleoporin-related//Dirigent protein	Stress defense response	0.92	37
**Sobic.005G101500**	Nucleoporin-related//Dirigent protein	Stress defense response	0.81	28
**Sobic.003G111200**	Potato type II proteinase inhibitor family	Stress defense response	0.93	28
**Sobic.005G169200**	Pathogenesis-related protein 4	Stress defense response	0.81	25
**Sobic.008G182900**	Pathogenesis related protein 5	Stress defense response	0.90	23
**Sobic.003G111300**	serine type endopeptidase inhibitor	Stress defense response	0.87	22
**Sobic.002G074400**	NAD(P)H:quinone oxidoreductase, WrbA	Redox homeostasis	0.90	25
**Sobic.002G004700**	NAD(P)H:quinone oxidoreductase, WrbA	Redox homeostasis	0.78	13
**Sobic.001G264000**	Alcohol dehydrogenase 2	Redox homeostasis	0.86	12
**Sobic.004G304300**	short-chain dehydrogenase/reductase	Redox homeostasis	0.82	9
**Sobic.005G082700**	Oxidoreductase, zinc-binding dehydrogenase family	Redox homeostasis	0.69	1

a Gene description obtained from Phytozome description based on Sbicolor_730_v5.0 (https://phytozome-next.jgi.doe.gov/info/Sbicolor_v5_1) reference genome.

b Function based on literature search for gene model.

In examining the flavonoid biosynthetic pathway in sorghum pericarp (**Figure 5**), unique gene family members at individual steps in the pathway showed moderate-to-strong correlations with pericarp LUT (the predominant sorghum pericarp 3-DOA), and many of these gene family members also clustered into the Darkgrey module in the combined WGCNA analysis. Early biosynthetic pathway genes in the Darkgrey module that correlated with LUT content included the following gene family members: phenylalanine ammonia-lyase (PAL) Sobic.004G220300 (r=0.58); cinnamate-4-hydroxylase (C4H) Sobic.002G126600 (r=0.55); and 4-coumarate:CoA ligase (4CL) Sobic.004G062500 (r=0.45). The first dedicated step in flavonoid biosynthesis is catalyzed by CHS, and two family members, Sobic.005G136300 (r=0.95) and Sobic.005G137015 (r=0.71), were highly correlated with LUT content (**Figure 5**). Three CHS gene models (Sobic.005G136300, r=0.95; Sobic.005G137133, r=0.49; and Sobic.005G137200, r=0.55) represented hub genes in the Darkgrey module (**Table 1**). Naringenin chalcone is then converted by CHI to naringenin, and CHI gene model Sobic.001G035600 (r=0.79) exhibited the strongest association with pericarp 3-DOA biosynthesis. Naringenin is converted to an intermediate flavone eriodictyol by an F3’H, of which hub gene Sobic.004G200788 was found to be the gene family member most strongly associated with LUT levels in black pericarp (r=0.83). This F3’H hub gene is also located in a locus previously reported to be associated with wounding and pathogen-induced 3-DOA biosynthesis in sorghum vegetative tissue (Mizuno et al., 2014). The final ill-defined steps in the 3-DOA biosynthetic pathway are proposed to involve flavone 4-reductases (FNRs) and/or DFRs that convert naringenin to flavan-4-ols, apiforol and luteoforol (Liu et al., 2010; Kawahigashi et al., 2016; Mizuno et al., 2016). Hub genes Sobic.004G050200 (r=0.92) and Sobic.009G043800 (r=0.90) were two DFR/FNR gene family members that were highly correlated with pericarp LUT levels. In addition, the FNR identified as the *P* gene (Sobic.006G226800) that has been associated with leaf color changes upon wounding of sorghum leaves was a highly connected hub gene with a strong association (r=0.92) with pericarp LUT levels in response to high-fluence UV. The highly connected hub gene Sobic.002G000400 (r=0.95) that correlates strongly with 3-DOA levels has been reported to be a flavone synthase II (FNSII), which represents an alternative branch point from naringenin to the flavones apigenin and luteolin (Mizuno et al., 2016) (**Figure 5**, **Table 1**). In addition to the well-defined structural genes in the flavonoid pathway, other hub genes potentially involved in 3-DOA biosynthesis were *O*-methyltransferases (Sobic.010G231000, r=0.99; and Sobic.007G058800, r=0.95), and *O*-glucosyl and galactosyl transferases (Sobic.010G238400, r=0.94; and Sobic.004G190800, r=0.93). It should be noted that a cinnamoyl-CoA reductase (Sobic.007G141200, r=0.94) was also identified as a hub gene in the Darkgrey module despite being annotated as the first committed enzyme of the lignin branch biosynthetic pathway.

In addition to detecting hub genes that represent structural genes in the flavonoid pathway, a series of hub genes involved in cellular transport were identified (**Table 1**). These hub genes included several highly connected ABC transporters (Sobic.003G216000, r=0.93; and Sobic.003G216232, r=0.72), GST (Sobic.005G212700, r=0.85) and a panel of additional transporters implicated in metabolite transport within plant cells (**Supplementary Table 2**).

#### 3.5.2 Stress defense response and redox homeostasis

A series of hub genes involved in stress defense response and redox homeostasis were detected (**Table 1**). Hub genes correlated with 3-DOA levels in black pericarp under UV included a suite of pathogen-related proteins (e.g., Sobic.008G182900, r=0.90; Sobic.001G401300, r=0.88; and Sobic.005G169200, r=0.81), nucleoporin-related dirigent proteins (Sobic.005G101500, r= 0.81; Sobic.005G101800 r= 0.92; and Sobic.005G101600, r=0.89), and serine type endopeptidase (protease) inhibitors (e.g., Sobic.003G111300, r=0.87; and Sobic.003G111200, r=0.93), amongst others. Five Darkgrey module hub genes involved in redox homeostasis included several dehydrogenases/reductases (Sobic.001G264000, r=0.86; Sobic.004G304300, r=0.82; and Sobic.005G082700, r=0.69), and two NAD(P)H:quinone oxidoreductases (WrbA; Sobic.002G074400, r=0.90; and Sobic.002G004700, r=0.78). NAD(P)H:quinone oxidoreductases catalyze the two-electron reduction of quinone compounds to prevent the generation of ROS in mammalian systems (Anusevicius et al., 2002), and these redox homeostasis enzymes have been extensively studied in animal systems for their physiological role in the reduction of free radical load in cells and the detoxification of xenobiotics (Lee et al., 2021). Finally, while not identified as hub genes, the Darkgrey module contained genes involved in redox homeostasis including catalase isozyme 3 (Sobic.004G011566, r=0.44) and five members of the GST family (Sobic.005G212700, r=0.85; Sobic.008G074400, r=0.64; Sobic.003G426500, r=0.51; Sobic.001G317200, r=0.48; and Sobic.001G012500, r=0.45) that can be found in the full results of the WGCNA analysis in **Supplementary Data Sheet 5**.

#### 3.5.3 Cellular signaling

A series of hub genes with strong association with pericarp LUT levels were identified including a group of protein kinase domain-containing genes (**Table 1**). In particular, a number of receptor-like kinase (RLK) hub genes (Sobic.001G074800, r=0.86; Sobic.002G024100, r=0.84; Sobic.002G024300, r=0.85; and Sobic.006G229600, r=0.78) were associated with pericarp LUT biosynthesis, as were several proteins implicated in phytohormone signaling including an auxin induced-like protein (Sobic.006G208300, r=0.85) and a putative brassinosteroid-insensitive associated receptor kinase (Sobic.005G126200, r=0.87). Finally, a particular leucine-rich repeat (LRR) gene (Sobic.009G012900, r=0.97) was strongly associated with pericarp LUT content and represented a hub gene with extensive intramodular connectivity.

#### 3.5.4 Transcription factors

Several TFs were identified as hub genes with multiple connections within the Darkgrey module (**Table 1**). Amongst the TFs identified strongly correlated with 3-DOA levels and highly connected (co-expressed) were NAC domain TFs (Sobic.001G071000, r=0.95; and Sobic.008G183900, r=0.88) and a bHLH TF family member (Sobic.003G031100, r=0.97). Also, highly correlated with 3-DOAs were several WRKY gene models including a TF with homology to WRKY72 in Arabidopsis (Sobic.010G035300, r=0.90) and a second putative WRKY TF Sobic.009G234100 (r=0.73). Finally, two C2H2-type Zinc finger containing genes (Sobic.003G349600, r=0.88; and Sobic.009G164600, r=0.60) represent hub genes in the Darkgrey module. While not representing hub genes, additional TFs clustered in the Darkgrey module including MYB TFs (Sobic.008G055700, r=0.59; Sobic.002G423300, r=0.48; Sobic.002G337800, r=0.36; and Sobic.003G267400, r=0.26), a WD repeat domain TF (Sobic.003G416400, r=0.47), and an AP2/ethylene responsive element TF (Sobic.004G296900, r=0.70) that can be found in the full results of the WGCNA analysis in **Supplementary Data Sheet 5**.

#### 3.5.5 *Cis*-regulatory Elements

The promoter regions of the Darkgrey (highly correlated with LUT levels) and the Saddlebrown (negatively correlated with LUT levels) gene modules were subjected to a DNA-binding motif enrichment analysis with the motif discovery program MEME. The promoter regions (2 kb upstream from the transcriptional start site) for all genes within these two clusters were significantly enriched (*p*-value < 0.01) for a Growth Regulation Factor (GRF) family TF binding site, specifically GRF4 (Kuijt et al., 2014). When sub-setting these clusters for the top correlated or negatively correlated genes to LUT, additional TF recognition domains were revealed. The promoter regions for the 138 strongest correlated genes in the Darkgrey module (LUT correlation ≥ 0.60) were enriched for MYB and GARP (Golden2, ARR-B, Psr1) family TF binding sites (**Supplementary Data Sheet 9**). Specifically, 65 of the 138 promoters had the MYB *cis* regulatory element (CRE) while 37 promoters contained a GARP motif. Notably, the 3-DOA associated gene *Y1* is also a MYB TF (Boddu et al., 2005) while the GARP motif showed strong cross recognition for ARR-B and HHO5 TFs, whose orthologs have been associated with floral development and environmental response cues (Moreau et al., 2016; Li et al., 2021). The genes that contained the MYB motif were enriched for biological process ontologies including those related to phenylpropanoid biosynthesis (GO:0009698), oxidation-reduction process (GO:0055114), single-organism metabolic process (GO:0044710), and response to UV-B (GO:0010224), whereas the GARP motif-containing promoters were upstream of genes involved in response to karrikin (GO:0080167) and flavonoid metabolic process (GO:0009812) (**Table 2**). The promoter regions for 70 of the 124 strongest negatively correlated genes (LUT correlation ≤ -0.25) in the Saddlebrown cluster yielded significant enrichment for a BARLEY B RECOMBINANT/BASIC PENTACYSTEINE (BBR/BPC) family binding site Class I BASIC PENTACYSTEINE1 (BPC1), which is also identical to a RAMOSA1 binding site (**Supplementary Data Sheet 9**).

**Table 2:** TF motif enrichment analysis and associated GO terms of Darkgrey WGCNA module.

Motif	GO ID	Term	Genes
MYB	GO:0019748	secondary metabolic process	Sobic.001G235500, Sobic.002G000400, Sobic.003G264500, Sobic.005G101500, Sobic.005G101700, Sobic.005G101800, Sobic.005G136300, Sobic.007G141200, Sobic.010G230900
	GO:0009698	phenylpropanoid metabolic process	Sobic.005G101500, Sobic.005G101700, Sobic.005G101800, Sobic.005G136300, Sobic.007G141200
	GO:0050790	regulation of catalytic activity	Sobic.001G401300, Sobic.002G103800, Sobic.003G111300, Sobic.005G101500, Sobic.005G101700, Sobic.005G101800
	GO:0065009	regulation of molecular function	Sobic.001G401300, Sobic.002G103800, Sobic.003G111300, Sobic.005G101500, Sobic.005G101700, Sobic.005G101800
	GO:0044710	single-organism metabolic process	Sobic.001G005700, Sobic.001G235500, Sobic.001G407800, Sobic.001G453100, Sobic.002G000400, Sobic.002G103800, Sobic.003G264500, Sobic.003G356000, Sobic.005G101500, Sobic.005G101700, Sobic.005G101800, Sobic.005G136300, Sobic.006G126300, Sobic.006G181300, Sobic.006G220500, Sobic.007G141200, Sobic.008G074400, Sobic.010G042300, Sobic.010G071800, Sobic.010G092200, Sobic.010G230900
	GO:0055114	oxidation-reduction process	Sobic.001G235500, Sobic.001G407800, Sobic.002G000400, Sobic.002G103800, Sobic.003G356000, Sobic.006G126300, Sobic.006G181300, Sobic.006G220500, Sobic.010G071800, Sobic.010G092200, Sobic.010G230900
	GO:0010224	response to UV-B	Sobic.001G035600, Sobic.005G136300
GARP ARR-B/HH05	GO:0009812	flavonoid metabolic process	Sobic.001G035600, Sobic.003G047500, Sobic.007G223900
	GO:0080167	response to karrikin	Sobic.001G035600, Sobic.001G285400
NAC	GO:0009812	flavonoid metabolic process	Sobic.001G035600, Sobic.003G047500, Sobic.007G223900
GRF4	GO:0006560	proline metabolic process	Sobic.001G071000, Sobic.003G356000
	GO:0019748	secondary metabolic process	Sobic.001G071000, Sobic.001G235500, Sobic.002G000400, Sobic.003G203500, Sobic.003G264500, Sobic.005G101500, Sobic.005G101700
	GO:0009064	glutamine family amino acid metabolic process	Sobic.001G071000, Sobic.001G451500, Sobic.003G356000
	GO:0008652	cellular amino acid biosynthetic process	Sobic.001G071000, Sobic.001G451500, Sobic.001G453100, Sobic.003G356000
	GO:0009812	flavonoid metabolic process	Sobic.001G071000, Sobic.003G047500, Sobic.007G223900, Sobic.010G238400

## 4 Discussion

The black grain trait in sorghum has notable value in the specialty food and nutraceutical industries owing to the biosynthesis of 3-DOAs, a group of polyphenols not commonly present in higher plants (Awika et al., 2004). These polyphenols are unique from more common anthocyanins in that 3-DOAs are more stable in aqueous solutions. This stability is valued for use as natural food colorants, organic food preservatives, and antioxidant food additives. While the biosynthesis of 3-DOAs in sorghum has been observed in both vegetative and pericarp tissue, the environmental signals and genetic mechanisms that condition 3-DOA biosynthesis are genotype- and organ-specific. Accumulation of 3-DOAs in vegetative tissues is a common and dominant trait with the induction of 3-DOA biosynthesis occurring in response to pathogen/insect attack or mechanical wounding (Lo et al., 1999; Nielsen et al., 2004; Mizuno et al., 2014; Kawahigashi et al., 2016). In contrast, the inheritance of 3-DOA biosynthesis in pericarp of black sorghum is complex (recessive with significant additive, dominant, and epistatic effects), and the accumulation of pericarp 3-DOAs is in response to the environmental stress associated with high-fluence UVB (Pfeiffer and Rooney, 2016; Fedenia et al., 2020). These initial genetic studies of black pericarp sorghum and the associated accumulation of 3-DOAs revealed the complex inheritance of this trait and the metabolic pathways upregulated in black pericarp cells in response to UVB light. The present study attempted to identify key biosynthetic and regulatory genes of 3-DOA accumulation in black pericarp sorghum while gaining a deeper understanding of the gene networks and cellular events controlling this tissue- and genotype-specific trait.

### 4.1 Temporal gene expression in black pericarp and metabolic reprogramming

Lo and Nicholson (1998) reported that sorghum mesocotyls repress various metabolic activities (such as anthocyanin synthesis) to compensate for the immediate need for defense responses including the biosynthesis of 3-DOAs as phytoalexins. In the present study, the temporal changes in gene expression in black vs. red pericarp revealed reprogramming of cellular metabolism of pericarp tissues throughout grain maturation under UV stress. GO enrichment in black pericarp revealed the activation of genes related to abiotic stimulus, secondary metabolic processes, glutathione metabolism, flavonoid biosynthesis, and flavonoid glucuronidation (amongst others) across the entire time series (**Supplementary Data Sheet 2**). DEGs pertaining to response to UV, response to ROS, response to wounding, and lignin metabolism (amongst others) are enriched in maturing black grain (10-17 DAA). In addition, GO enrichment indicated the downregulation in black pericarp under UV of genes involved in primary metabolic, cellular, nucleic acid metabolic processes, and regulation of gene expression (epigenetic, posttranscriptional). The results demonstrate that the pericarp of black grain perceives ambient UV as an environmental stress throughout grain development, which is reflected in the marked reprogramming of gene expression in pericarp cells leading to the biosynthesis of defense-related metabolites including 3-DOAs.

### 4.2 Functional divergence of flavonoid pathway multi-gene families

The duplication and subsequent functional diversification or pseudogenization of multi-gene family members is a common evolutionary process, and this process has occurred in both the early and late biosynthetic genes in flavonoid biosynthesis in sorghum (Shih et al., 2006; Liu et al., 2010). Within sorghum, the early biosynthetic pathway genes represent multigene families with specific family members being upregulated in sorghum in response to specific abiotic (Liu et al., 2010; Kawahigashi et al., 2016) or biotic (Mizuno et al., 2012, 2014; Pant and Huang, 2022) stressors in leaves and developing grain. In pericarp of black sorghum, certain early biosynthetic pathway gene family members were moderately correlated (0.45 ≥ r ≤ 0.58) with 3-DOA levels in pericarp, and some of these gene family members have been reported to be upregulated in leaves in response to pathogen (Liu et al., 2010; Mizuno et al., 2012) and insect attack/wounding (Mizuno et al., 2012; Pant and Huang, 2022). In the case of CHS, one gene model (Sobic.005G136300; alias Sb05g020160) was highly correlated (r=0.95) with black pericarp 3-DOA levels. This same gene model was previously reported to be induced by pathogen infestation in sorghum leaves (Liu et al., 2010). Four bi-functional DFR/FNR genes are annotated in the BTx623 v5.1 genome (https://phytozome-next.jgi.doe.gov/info/Sbicolor_v5_1). In response to fungal hyphae penetration of sorghum vegetative tissue, Sobic.004G050200 was upregulated and was associated with the localized biosynthesis of 3-DOAs (Liu et al., 2010). Similarly, UV irradiation of black pericarp also activated the expression of this hub gene. Thus, while this DFR family member is associated with the activation of 3-DOA biosynthesis, it is not specific to either vegetative or reproductive tissues nor is it specific to UV or pathogen stressors. By contrast, Sobic.009G043800 (alias DFR4) was not associated with 3-DOA biosynthesis in vegetative tissue under pathogen attack (Liu et al., 2010), but was a hub gene with high intramolecular connectivity and was highly correlated (r= 0.90) with the accumulation of 3-DOAs in black pericarp under UV stress (**Table 1**). In addition, this DFR was upregulated over 3,000-fold in black sorghum when compared to red pericarp sorghum at 17 DAA (**Supplementary Data Sheet 1**).

The *P* gene, Sobic.006G226800 (alias Sb06g029550), has been reported to be highly expressed in leaf tissues that accumulate 3-DOAs upon wounding (Mizuno et al., 2016), and this same gene model was also highly correlated with 3-DOA levels in black pericarp. Despite being implicated in the biosynthesis of flavon-4-ols, the present results may support the assertion that the *P* gene product may have overlapping activity between FNR and DFR (Lewis et al., 2023). Similarly, the strong correlation of FNSII (Sobic.002G000400, r=0.95) with pericarp LUT levels may be indicative of unquantified flavone levels in the pericarp of black sorghum under the present light regimes; alternatively, the gene product of Sobic.002G000400 may have an unknown enzymatic function in pericarp specific 3-DOA biosynthesis as it has been reported to be induced upon color changes in leaf tissue upon wounding or pathogen attack (Kawahigashi et al., 2016).

Late steps in 3-DOA biosynthesis revealed specific gene family members associated with pericarp 3-DOA accumulation. F3’H converts naringenin to intermediate eriodictyol (Shih et al., 2006). F3’H Sobic.004G200788 (Sobic.004G200800v3; alias Sb04g024710) is a hub gene in the Darkgrey module (r=0.83) (**Table 1**) and differentially expressed at 17 DAA only (106-fold) in RTx3362 vs BTx378 pericarp tissue (**Supplementary Data Sheet 1**). An additional F3’H, Sobic.004G200744 (Sobic.004G200833v3, r=0.77), used as a bait gene, was highly correlated to total 3-DOA levels and LUT under experiment 1 (r=0.995), and represented a hub gene in the combined WGCNA analysis (**Table 1**, **Supplementary Data Sheet 5**). Five F3’H genes are annotated on chromosome 4 (57,703,680- 57,762,066 kb) in the BTx623v5.1 genome, and previous reports (Mizuno et al., 2016) provide evidence of structural variations in these tandem arrayed genes. The present findings support the functional diversification of these tandem F3’H genes in black sorghum in a tissue-specific manner. Finally, of the relatively large gene family of glucosyl and galactosyl transferases within sorghum, three gene models (Sobic.010G238400, Sobic.004G190800, and Sobic.007G223900) were hub genes that were highly correlated with 3-DOA accumulation in black pericarp (**Table 1**). In addition, two hub genes annotated as methyltransferases (Sobic.010G231000 and Sobic.007G058800) were also highly associated with black pericarp 3-DOA levels. The co-expression of these diverse transferases with the biosynthesis of 3-DOAs presented here may guide future studies to elucidate the final steps in the biosynthesis of these flavon-4-ols.

### 4.3 ROS and cell signaling cascade in UV-induced 3-DOA biosynthesis

**Figure 6:**
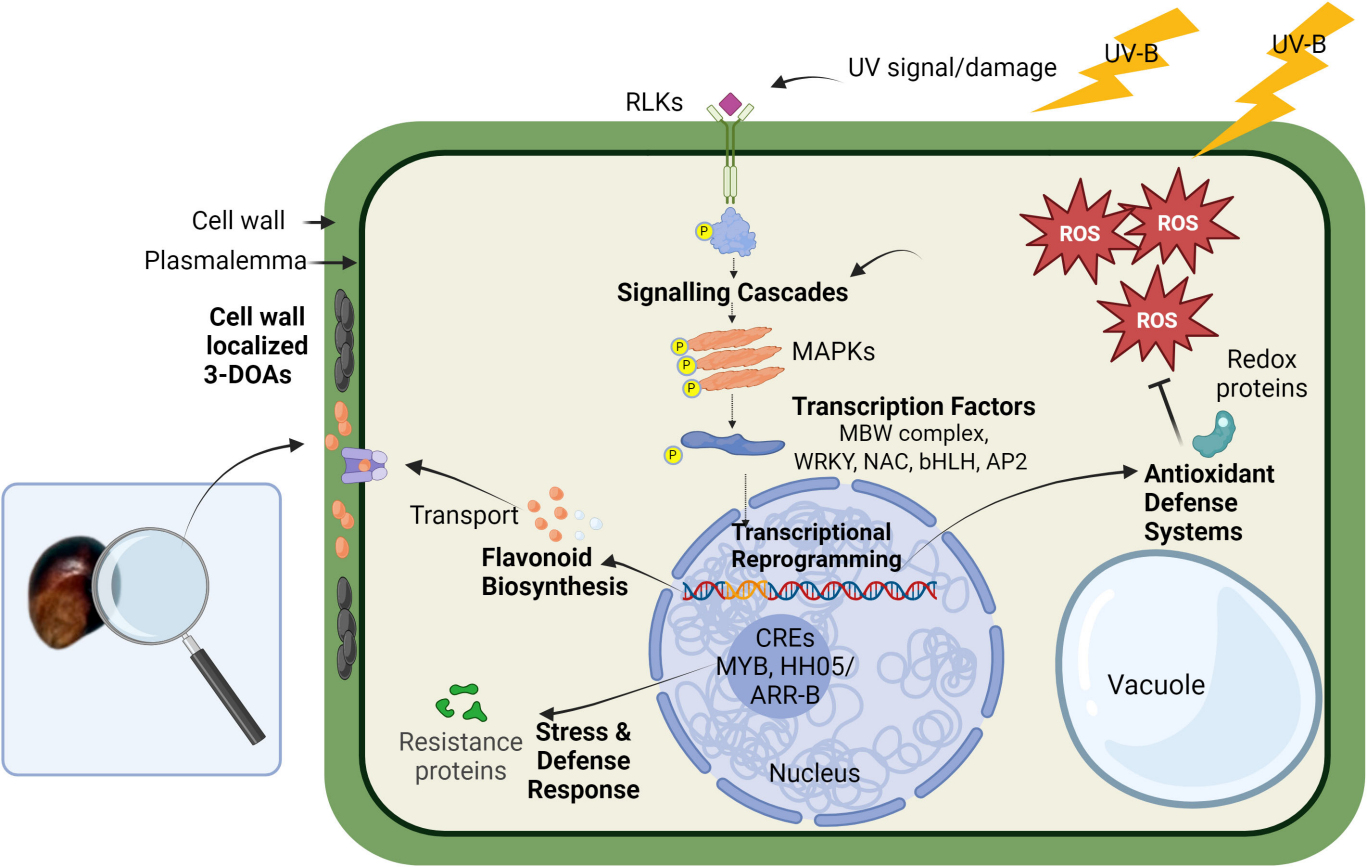
Hypothetical cellular model of UV-induced 3-DOA biosynthesis in pericarp of black sorghum. High-fluence UV exposure results in the generation of ROS that is proposed to play a key role in mediating downstream biological processes. The downstream processes are proposed to include kinase cascades and transcriptional activation of genes including TFs along with plant defense response genes such as resistance proteins, antioxidant systems, and conserved nucleotide-binding site–leucine-rich repeat proteins. Associated with these events is the transcriptional activation of structural genes in the biosynthetic pathway of 3-DOAs along with cellular transporters, which leads to accumulation of elevated levels of 3-DOAs in specific subcellular locations. Unlike anthocyanins that are localized in the vacuole, 3-DOAs are trafficked to the cell wall where they are proposed to serve as a protective sunshade against UV light.

Through an examination of cellular ROS levels, light microscopy of flavonoid localization in black pericarp tissue, and a gene network analysis of black pericarp 3-DOA biosynthesis, we propose a hypothetical cellular model of 3-DOA induction by UV in pericarp of black sorghum (**Figure 6**). This hypothetical model is proposed based on the present study, literature pertaining to the biosynthesis of 3-DOA phytoalexins in sorghum vegetative tissues, and the extensive body of literature related to plant defense responses to abiotic and biotic stresses. It should be noted that there is a general lack of functional verification of the role of specific genes and ROS in this signaling pathway. Given this fact, this model is provided as a framework for on-going studies to validate (or refute) the proposed cellular events associated with this rare, and value-added trait.

In the case of black sorghum, sustained exposure of pericarp during grain filling to full sunlight is perceived as an environmental stress that is required for full penetrance of black pericarp coloration and the associated accumulation of elevated 3-DOA levels. As is the case with 3-DOA biosynthesis in sorghum leaves in response to wounding or fungal attack, we propose that similar (yet unknown) cellular signals trigger 3-DOAs in black sorghum pericarp cells under high fluence UV. The present evidence suggests that ROS generated in pericarp cells exposed to full sunlight may be a component of the pathway and while the exact role of ROS signaling remains unknown, the signaling pathway in black pericarp under full sunlight appears to share features of various cellular signaling cascades of plant defense (Hou et al., 2019).

Distinct from elicitors derived from invading pathogens, several plant species can produce and recognize endogenous host-derived elicitor molecules (Yamaguchi and Huffaker, 2011). Endogenous elicitors, sometimes referred to as DAMPs, include ROS, oligosaccharide fragments, and protein fragments. Dixon (1986) introduced a model for phytoalexin biosynthesis, suggesting that endogenous elicitors produced by host cells act as signals to activate metabolic defense pathways. In signaling pathways endogenous elicitor molecules from either host or, in the case of fungal interaction, invader result in the deployment of a large number of RLKs and receptor-like proteins that often function at the cell surface during DAMP/PAMP triggered immunity (Tang et al., 2017; Hou et al., 2019). In black pericarp under high-fluence UV, it is unknown at present if an endogenous elicitor was derived from pericarp cells due to UV damage. What is known is that a series of receptor kinase hub genes were identified including leucine-rich repeat receptor kinases (Sobic.003G031100), lectin receptor-kinases (Sobic.001G074800, Sobic.002G024100, Sobic.002G024300, and Sobic.006G229600) and their co-receptor brassinosteroid insensitive 1-associated receptor kinase 1 (Sobic.005G126200). Downstream of receptor kinases, MAPKs are often linked to regulation of plant immunity and stress adaptation including adaptation to UV radiation and the associated redox state of the plant tissue (Raina et al., 2013; Hou et al., 2019). While the present network analysis did not identify MAPKs as critical hub genes, a series of MAPK gene models (Sobic.005G094301, Sobic.005G012200, Sobic.009G030600, Sobic.004G323600, Sobic.010G069600, and Sobic.002G299600) were transcriptionally activated at 17 DAA and some as early as 5 DAA (log_2_ fold change ≥ 1) in black pericarp under UV stress (**Supplementary Data Sheet 1**).

Downstream of the kinase cascades, a large family of TFs in plants are known to be involved in abiotic stress responses and in the regulation of flavonoid biosynthesis (Morishita et al., 2009; Ferreyra et al., 2012; Meraj et al., 2020). Although the classical regulation of flavonoid biosynthesis is mediated by the MBW complex (Zhuang et al., 2023), transcriptional regulation of the black sorghum grain trait also involved additional classes of TFs known to regulate stress-induced flavonoid biosynthesis (Wu et al., 2012; Yang et al., 2021). A series of TF hub genes were associated with the black pericarp phenotype including TF family members of the WRKY (Sobic.010G035300 and Sobic.009G234100; SbWRKY85), NAC (Sobic.008G183900 and Sobic.001G071000), Zinc finger C2H2 (Sobic.003G349600 and Sobic.009G164600) and a myc-type bHLH (Sobic.003G031100). These additional classes of TFs are known to regulate stress-induced flavonoid biosynthesis and may interact with the MBW complex or function upstream to regulate flavonoid biosynthesis (Wu et al., 2012; Yang et al., 2021). NAC TFs have been found as alternative regulators of UVB response apart from the known UVR8 pathway (O’Hara et al., 2019). NAC can also interact with MYBs to regulate anthocyanin biosynthesis in fruit (Zhang et al., 2020) as do bHLH TFs which are light responsive and can show differential regulation between tissues (Riaz et al., 2019). WRKY TFs are generally involved in abiotic stress responses and regulation of secondary metabolites (Meraj et al., 2020). The identification of these TFs with high correlation to 3-DOAs supports the complex overlapping stress and classical secondary metabolic regulatory cascades in the pericarp tissue of black sorghum under high-fluence UV. Despite the strong correlation between the transcriptional activation of TFs and the strong correlation with pericarp 3-DOA levels, how these TFs regulate (individually or in a complex) UV-induced 3-DOA biosynthesis and the cascade of subsequent cellular events leading to the black pigmentation of the grain are unknown.

DNA binding analysis of the promoter regions (2000 bp upstream of the TSS) of genes in the Darkgrey module revealed regulatory elements unique to genes correlated (r ≥ 0.60) to LUT that included MYB and HHO5/ARR-B motifs in the promoter regions (**Supplementary Data Sheet 9**). Hub genes with the MYB binding motif included many of the flavonoid biosynthesis genes in addition to numerous genes involved in defense response. Hub genes with the HHO5/ARR-B motif seemed less concentrated in particular ontologies, but included genes in the ROS, cell signaling, and regulatory TF modules. Both MYBs and NACs are large families of TFs involved in stress response in many plants, and the MYB Sobic.008G055700 has an established role in the regulation of secondary cell wall development, an important component of a plants defense system and barrier to attack (Hennet et al., 2020). The HHO5 transcriptional repressor is found in the promotor of NAC domain genes of which several were strongly correlated to LUT in the network (Sobic.008G183900, r=0.88; and Sobic.001G071000, r=0.95). HHO5 TFs are also suspected to be responsive to light, temperature, and hormone signals that influence floral organ development, seed germination, and flowering (Li et al., 2021). Altogether, these regulatory fingerprints further indicate that 3-DOA biosynthesis is tied to a general environmental stress; it also suggests the reprogramming in response to UV radiation within the pericarp of black sorghum (Mareya et al., 2019; Tugizimana et al., 2019). Finally, promoter analysis of the genes negatively correlated in the Saddlebrown module identified the plant specific BBR/BPC binding sites (same motif as RAMOSA1) in several genes correlated at r ≤ -0.25 to LUT (**Supplementary Data Sheet 9**). BBR/BPC TFs have diverse roles in developmental phase transitioning, hormone signaling, stress response, and seed development (Wu et al., 2020; Sahu et al., 2023). Reduced expression of this family of TFs resulted in a hypersensitive response in arabidopsis plants under salt stress and consequently recued growth, that may be an adaptive response to survive the stress (Yan et al., 2021; Sahu et al., 2023). The negative correlation of genes housing a BPC1 binding site is indicative of suppressed development likely due to a UV stress response.

In downstream responses to oxidative stress, plants are known to activate both enzymatic and non-enzymatic ROS scavenging systems to regulate cell redox homeostasis (Saed-Moucheshi et al., 2014; Zhuang et al., 2023). In black pericarp under high fluence UV, a panel of enzymatic antioxidant systems were transcriptionally activated including GST, alcohol dehydrogenase 2, and several hydrogenases (see **Table 1**). Of particular interest was the transcriptional activation of two WrBA gene family members (NAD(P)H:quinone oxidoreductases in black pericarp; the third WrBA family member (Sobic.004G185100) is deleted from the black sorghum genome (Fedenia, 2021). Quinone reductases have been extensively studied in mammalian systems, and their obligate two-electron reductions of quinone play critical roles in protection against oxidative stress and xenobiotic toxicity (Lee et al., 2021). While we hypothesize that the deletion of the WrBA gene family member may be central to the genetic inheritance of the black pericarp trait, this gene deletion is perhaps but one genetic signature of the complex inheritance of this quantitative trait.

Flavonoids also can act as an antioxidant agent and can directly scavenge ROS (Wu et al., 2023). In fruits and vegetables, the biosynthesis of flavonoids occurs in the cytoplasm and accumulates in vesicle-like structures that are finally transported by a series of enzymes including GST, ABC, and MATE transporters (Gomez et al., 2011). While anthocyanins are transported to the vacuole (Pourcel et al., 2010), 3-DOAs in sorghum leaf tissue under fungal attack have been reported to accumulate within the cytoplasm and are also complexed in the cell wall (Nielsen et al., 2004). The present light microscopy of sorghum pericarp appeared to show an increase of flavonoids associated with the cell wall in black pericarp tissue under full sunlight. This localization of 3-DOAs suggests possible reinforcement of the cell wall in pericarp surfaces directly exposed to UV. The fact that a significant portion of the 3-DOAs in black pericarp appear associated with the cell wall is supported by the well-known difficulty in extracting 3-DOAs from pericarp tissue, which requires microwave-assisted extraction (Herrman et al., 2020) or harsh acidic or alkaline extraction conditions (Stalikas, 2007) for quantitative extraction of 3-DOAs. It should be noted that the black pigmentation of the grain may act as a sunshade to impede UV from penetrating or the 3-DOAs may be functioning at a different cellular level in response to UV light. At present, it is unknown the exact cellular role (if any) of 3-DOAs protecting the grain of these sorghum genotypes from UV light.

## 5 Conclusion

The present WGCNA study provides insight into the changes in gene expression leading to the UV-dependent biosynthesis of 3-DOAs in pericarp of black sorghum. Illumination with full spectrum sunlight throughout grain maturation is associated with elevated ROS levels, activation of the biosynthesis of 3-DOAs, and the subcellular localization of flavonoids with the cell wall in pericarp of black sorghum. The changes in gene expression and activation of metabolic pathways in black pericarp to UV light parallels those cellular events associated with pathogen attack or mechanical wounding. The perception of UV light as a stress in black pericarp genotypes is not a common trait shared by red or white seeded sorghums. This study is based largely on correlations between 3-DOA measurements and gene expression across multiple biological and experimental replications that are complemented by cellular ROS determinations and subcellular localization of 3-DOAs. The inherent limitation of using co-expression for gene functional inferences for metabolic pathways requires consideration when interpreting the present results, and while functional validation is required to confirm the proposed cellular events associated with this pericarp-specific trait, transformation of sorghum is presently limited to white grained genotypes lacking 3-DOAs and functional alleles involved in this quantitatively inherited trait. Nevertheless, the information presented herein provides a framework for future genetic and cellular studies to validate the proposed cascade of events and the critical regulatory and structural genes involved in the sorghum black pericarp trait.

## Data Availability

The datasets generated for this study can be found in the https://www.ncbi.nlm.nih.gov/sra (bioproject PRJNA1072120) and at Github for WGCNA code: https://github.com/BrooklynSchu/WGCNA-Sorghum-Pericarp/blob/main/010324.

## References

[B1] Andrews S. (2010). FastQC: a quality control tool for high throughput sequence data. Available online at: http://www.bioinformatics.babraham.ac.uk/projects/fastqc/ (Accessed February 20, 2021).

[B2] Anusevicius Z. Sarlauskas J. Cenas N. (2002). Two-electron reduction of quinones by rat liver NAD(P)H:quinone oxidoreductase: quantitative structure-activity relationships. Arch. Biochem. Biophys. 404, 254–262. doi: 10.1016/s0003-9861(02)00273-4 12147263

[B3] Awika J. M. Rooney L. W. Waniska R. D. (2004). Properties of 3-deoxyanthocyanins from sorghum. J. Agric. Food Chem. 52, 4388–4394. doi: 10.1021/jf049653f 15237941

[B4] Awika J. M. Yang L. Y. Browning J. D. Faraj A. (2009). Comparative antioxidant, antiproliferative and phase II enzyme inducing potential of sorghum (*Sorghum bicolor*) varieties. Lwt-Food Sci. Technol. 42, 1041–1046. doi: 10.1016/j.lwt.2009.02.003

[B5] Bailey T. L. Johnson J. Grant C. E. Noble W. S. (2015). The MEME suite. Nucleic Acids Res. 43, W39–W49. doi: 10.1093/nar/gkv416 25953851 PMC4489269

[B6] Bates D. Mächler M. Bolker B. Walker S. (2015). Fitting linear mixed-effects models using lme4. J. Stat. Softw. 67, 1–48. doi: 10.18637/jss.v067.i01

[B7] Boddu J. Svabek C. Ibraheem F. Jones A. D. Chopra S. (2005). Characterization of a deletion allele of a sorghum Myb gene *yellow seed1* showing loss of 3-deoxyflavonoids. Plant Sci. 169, 542–552. doi: 10.1016/j.plantsci.2005.05.007

[B8] Bolger A. M. Lohse M. Usadel B. (2014). Trimmomatic: A flexible trimmer for Illumina sequence data. Bioinformatics 30, 2114–2120. doi: 10.1093/bioinformatics/btu170 24695404 PMC4103590

[B9] Checker V. G. Kushwaha H. R. Kumari P. Yadav S. (2018). “Role of phytohormones in plant defense: signaling and cross talk,” in Molecular Aspects of Plant-Pathogen Interaction. Ed. Singh A. I. (Springer, Singapore), 159–184.

[B10] Das K. Roychoudhury A. (2014). Reactive oxygen species (ROS) and response of antioxidants as ROS-scavengers during environmental stress in plants. Front. Env. Sci-Switz 2. doi: 10.3389/fenvs.2014.00053

[B11] Dias M. C. Pinto D. C. G. A. Silva A. M. S. (2021). Plant flavonoids: Chemical characteristics and biological activity. Molecules 26, 5377. doi: 10.3390/molecules26175377 34500810 PMC8434187

[B12] Dixon R. A. (1986). The phytoalexin response: Elicitation, signalling, and control of host gene expression. Biol. Rev. 61, 239–291. doi: 10.1111/j.1469-185X.1986.tb00719.x

[B13] Dykes L. Rooney L. W. (2007). Phenolic compounds in cereal grains and their health benefits. Cereal Foods World 52, 105–111. doi: 10.1094/Cfw-52-3-0105

[B14] Dykes L. Rooney W. L. Rooney L. W. (2013). Evaluation of phenolics and antioxidant activity of black sorghum hybrids. J. Cereal Sci. 58, 278–283. doi: 10.1016/j.jcs.2013.06.006

[B15] Dykes L. Rooney L. W. Waniska R. D. Rooney W. L. (2005). Phenolic compounds and antioxidant activity of sorghum grains of varying genotypes. J. Agr. Food Chem. 53, 6813–6818. doi: 10.1021/jf050419e 16104804

[B16] Dykes L. Seitz L. M. Rooney W. L. Rooney L. W. (2009). Flavonoid composition of red sorghum genotypes. Food Chem. 116, 313–317. doi: 10.1016/j.foodchem.2009.02.052 25214345

[B17] Fedenia L. (2021). Environmental and genetic control of black pericarp traits in sorghum (*Sorghum bicolor*): [A Dissertation]. Texas A&M University, College Station TX.

[B18] Fedenia L. Klein R. R. Dykes L. Rooney W. L. Klein P. E. (2020). Phenotypic, phytochemical, and transcriptomic analysis of black sorghum (*Sorghum bicolor* L.) pericarp in response to light quality. J. Agr. Food Chem. 68, 9917–9929. doi: 10.1021/acs.jafc.0c02657 32822185

[B19] Ferreyra M. L. F. Rius S. P. Casati P. (2012). Flavonoids: Biosynthesis, biological functions, and biotechnological applications. Front. Plant Sci. 3. doi: 10.3389/fpls.2012.00222 PMC3460232 23060891

[B20] Fornes O. Castro-Mondragon J. A. Khan A. van der Lee R. Zhang X. Richmond P. A.. (2020). Jaspar 2020: Update of the open-access database of transcription factor binding profiles. Nucleic Acids Res. 48, D87–D92. doi: 10.1093/nar/gkz1001 31701148 PMC7145627

[B21] Gomez C. Conejero G. Torregrosa L. Cheynier V. Terrier N. Ageorges A. (2011). *In vivo* grapevine anthocyanin transport involves vesicle-mediated trafficking and the contribution of anthoMATE transporters and GST. Plant J. 67, 960–970. doi: 10.1111/j.1365-313X.2011.04648.x 21605207

[B22] González-Montilla F. M. Chávez-Santoscoy R. A. Gutiérrez-Uribe J. A. Serna-Saldivar S. O. (2012). Isolation and identification of phase II enzyme inductors obtained from black Shawaya sorghum [*Sorghum bicolor* (L.) Moench] bran. J. Cereal Sci. 55, 126–131. doi: 10.1016/j.jcs.2011.10.009

[B23] Grant C. E. Bailey T. L. Noble W. S. (2011). FIMO: Scanning for occurrences of a given motif. Bioinformatics 27, 1017–1018. doi: 10.1093/bioinformatics/btr064 21330290 PMC3065696

[B24] Guo J. Han W. Wang M. (2008). Ultraviolet and environmental stresses involved in the induction and regulation of anthocyanin biosynthesis: A review. Afr. J. Biotechnol. 7, 4966–4972. doi: 10.5897/AJB08.090

[B25] Gupta S. Stamatoyannopoulos J. A. Bailey T. L. Noble W. S. (2007). Quantifying similarity between motifs. Genome Biol. 8, R24. doi: 10.1186/gb-2007-8-2-r24 17324271 PMC1852410

[B26] Hahn D. H. Rooney L. W. Earp C. F. (1984). Tannins and phenols of sorghum. Cereal Food World 29, 776–779.

[B27] Han Z. G. Ahsan M. Adil M. F. Chen X. H. Nazir M. M. Shamsi I. H.. (2020). Identification of the gene network modules highly associated with the synthesis of phenolics compounds in barley by transcriptome and metabolome analysis. Food Chem. 323, 126862. doi: 10.1016/j.foodchem.2020.126862 32334297

[B28] Hennet L. Berger A. Trabanco N. Ricciuti E. Dufayard J.-F. Bocs S.. (2020). Transcriptional regulation of sorghum stem composition: Key players identified through co-expression gene network and comparative genomics analyses. Front. Plant Sci. 11. doi: 10.3389/fpls.2020.00224 PMC7064007 32194601

[B29] Herrman D. A. Brantsen J. F. Ravisankar S. Lee K.-M. Awika J. M. (2020). Stability of 3-deoxyanthocyanin pigment structure relative to anthocyanins from grains under microwave assisted extraction. Food Chem. 333, 127494. doi: 10.1016/j.foodchem.2020.127494 32663754

[B30] Hou S. G. Liu Z. Y. Shen H. X. Wu D. J. (2019). Damage-associated molecular pattern-triggered immunity in plants. Front. Plant Sci. 10. doi: 10.3389/fpls.2019.00646 PMC6547358 31191574

[B31] Ibraheem F. Gaffoor I. Chopra S. (2010). Flavonoid phytoalexin-dependent resistance to anthracnose leaf blight requires a functional *yellow seed1* in *Sorghum bicolor* . Genetics 184, 915–926. doi: 10.1534/genetics.109.111831 20083611 PMC2865927

[B32] Isah T. (2019). Stress and defense responses in plant secondary metabolites production. Biol. Res. 52, 39. doi: 10.1186/s40659-019-0246-3 31358053 PMC6661828

[B33] Jin J. Tian F. Yang D. C. Meng Y. Q. Kong L. Luo J.. (2017). PlantTFDB 4.0: Toward a central hub for transcription factors and regulatory interactions in plants. Nucleic Acids Res. 45, D1040–D1045. doi: 10.1093/nar/gkw982 27924042 PMC5210657

[B34] Kawahigashi H. Kasuga S. Sawada Y. Yonemaru J.-I. Ando T. Kanamori H.. (2016). The sorghum gene for leaf color changes upon wounding (*P*) encodes a flavanone 4-reductase in the 3-deoxyanthocyanidin biosynthesis pathway. G3-Genes Genom. Genet. 6, 1439–1447. doi: 10.1534/g3.115.026104 PMC4856094 26994288

[B35] Kuijt S. J. H. Greco R. Agalou A. Shao J. t Hoen C. C. J. Övernäs E.. (2014). Interaction between the GROWTH-REGULATING FACTOR and KNOTTED1-LIKE HOMEOBOX families of transcription factors. Plant Physiol. 164, 1952–1966. doi: 10.1104/pp.113.222836 24532604 PMC3982755

[B36] Langfelder P. Horvath S. (2008). WGCNA: An R package for weighted correlation network analysis. BMC Bioinf. 9, 599. doi: 10.1186/1471-2105-9-559 PMC2631488 19114008

[B37] Langfelder P. Horvath S. (2012). Fast R functions for robust correlations and hierarchical clustering. J. Stat. Software 46, 1–17. doi: 10.18637/jss.v046.i11 PMC3465711 23050260

[B38] Lee W.-S. Ham W. Kim J. (2021). Roles of NAD(P)H:quinone oxidoreductase 1 in diverse diseases. Life 11, 1301. doi: 10.3390/life11121301 34947831 PMC8703842

[B39] Lewis J. A. Zhang B. Harza R. Palmer N. Sarath G. Sattler S. E.. (2023). Structural similarities and overlapping activities among dihydroflavonol 4-reductase, flavanone 4-reductase, and anthocyanidin reductase offer metabolic flexibility in the flavonoid pathway. Int. J. Mol. Sci. 24, 13901. doi: 10.3390/ijms241813901 37762209 PMC10531346

[B40] Li C. Chen L. Fan Q. He P. Wang C. Huang H.. (2023). Weighted gene co-expression network analysis to explore hub genes of resveratrol biosynthesis in exocarp and mesocarp of ‘summer black’ grape. Plants 12, 578. doi: 10.3390/plants12030578 36771662 PMC9920568

[B41] Li T. Wang Y. Dong Q. Wang F. Kong F. Liu G.. (2022). Weighted gene co-expression network analysis reveals key module and hub genes associated with the anthocyanin biosynthesis in maize pericarp. Front. Plant Sci. 13. doi: 10.3389/fpls.2022.1013412 PMC9661197 36388502

[B42] Li Q. Zhou L. Li Y. Zhang D. Gao Y. (2021). Plant NIGT1/HRS1/HHO transcription factors: Key regulators with multiple roles in plant growth, development, and stress responses. Int. J. Mol. Sci. 22, 3390. doi: 10.3390/ijms22168685 34445391 PMC8395448

[B43] Liu H. Du Y. Chu H. Shih C. H. Wong Y. W. Wang M.. (2010). Molecular dissection of the pathogen-inducible 3-deoxyanthocyanidin biosynthesis pathway in sorghum. Plant Cell Physiol. 51, 1173–1185. doi: 10.1093/pcp/pcq080 20529887

[B44] Lo S.-C. C. Hipskind J. D. Nicholson R. L. (1999). cDNA cloning of a sorghum pathogenesis-related protein (PR-10) and differential expression of defense-related genes following inoculation with *Cochliobolus heterostrophus* or *Colletotrichum sublineolum* . Mol. Plant Microbe Interact. 12, 479–489. doi: 10.1094/MPMI.1999.12.6.479 10356799

[B45] Lo S.-C. C. Nicholson R. L. (1998). Reduction of light-induced anthocyanin accumulation in inoculated sorghum mesocotyls. Implications for a compensatory role in the defense response. Plant Physiol. 116, 979–989. doi: 10.1104/pp.116.3.979 9501130 PMC35099

[B46] Mandal S. Ji W. M. McKnight T. D. (2020). Candidate gene networks for acylsugar metabolism and plant defense in wild tomato *Solanum pennellii* . Plant Cell. 32, 81–99. doi: 10.1105/tpc.19.00552 31628166 PMC6961621

[B47] Mandal S. Rezenom Y. H. McKnight T. D. (2022). ASTF1, an AP2/ERF-family transcription factor and ortholog of cultivated tomato LEAFLESS, is required for acylsugar biosynthesis. Available online at: https://www.biorxiv.org/content/10.1101/2022.04.04.487036v1 (Accessed May 1, 2024).

[B48] Mareya C. R. Tugizimana F. Piater L. A. Madala N. E. Steenkamp P. A. Dubery I. A. (2019). Untargeted metabolomics reveal defensome-related metabolic reprogramming in *Sorghum bicolor* against infection by *Burkholderia andropogonis* . Metabolites 9, 8. doi: 10.3390/metabo9010008 30609758 PMC6359421

[B49] Meraj T. A. Fu J. Raza M. A. Zhu C. Shen Q. Xu D.. (2020). Transcriptional factors regulate plant stress responses through mediating secondary metabolism. Genes 11, 346. doi: 10.3390/genes11040346 32218164 PMC7230336

[B50] Mizuno H. Kawahigashi H. Kawahara Y. Kanamori H. Ogata J. Minami H.. (2012). Global transcriptome analysis reveals distinct expression among duplicated genes during sorghum-*Bipolaris sorghicola* interaction. BMC Plant Biol. 12, 121. doi: 10.1186/1471-2229-12-121 22838966 PMC3480847

[B51] Mizuno H. Yazawa T. Kasuga S. Sawada Y. Kanamori H. Ogo Y.. (2016). Expression of flavone synthase II and flavonoid 3’-hydroxylase is associated with color variation in tan-colored injured leaves of sorghum. Front. Plant Sci. 7. doi: 10.3389/fpls.2016.01718 PMC5116553 27917182

[B52] Mizuno H. Yazawa T. Kasuga S. Sawada Y. Ogata J. Ando T.. (2014). Expression level of a flavonoid 3’-hydroxylase gene determines pathogen-induced color variation in sorghum. BMC Res. Notes 7, 1–12. doi: 10.1186/1756-0500-7-761 25346182 PMC4219097

[B53] Moreau F. Thévenon E. Blanvillain R. Lopez-Vidriero I. Franco-Zorrilla J. M. Dumas R.. (2016). The Myb-domain protein ULTRAPETALA1 INTERACTING FACTOR 1 controls floral meristem activities in *Arabidopsis* . Development 143, 1108–1119. doi: 10.1242/dev.127365 26903506

[B54] Morishita T. Kojima Y. Maruta T. Nishizawa-Yokoi A. Yabuta Y. Shigeoka S. (2009). Arabidopsis NAC transcription factor, ANAC078, regulates flavonoid biosynthesis under high-light. Plant Cell Physiol. 50, 2210–2222. doi: 10.1093/pcp/pcp159 19887540

[B55] Naik J. Misra P. Trivedi P. K. Pandey A. (2022). Molecular components associated with the regulation of flavonoid biosynthesis. Plant Sci. 317, 111196. doi: 10.1016/j.plantsci.2022.111196 35193745

[B56] Nida H. Lee S. Li Y. Mengiste T. (2021). Transcriptome analysis of early stages of sorghum grain mold disease reveals defense regulators and metabolic pathways associated with resistance. BMC Genomics 22, 295. doi: 10.1186/s12864-021-07609-y 33888060 PMC8063297

[B57] Nielsen K. A. Gotfredsen C. H. Buch-Pedersen M. J. Ammitzbøll H. Mattsson O. Duus J. Ø.. (2004). Inclusions of flavonoid 3-deoxyanthocyanidins in *Sorghum bicolor* self-organize into spherical structures. Physiol. Mol. Plant Pathol. 65, 187–196. doi: 10.1016/j.pmpp.2005.02.001

[B58] Nignpense B. E. Francis N. Blanchard C. Santhakumar A. B. (2021). Bioaccessibility and bioactivity of cereal polyphenols: A review. Foods 10, 1595. doi: 10.3390/foods10071595 34359469 PMC8307242

[B59] O’Hara A. Headland L. R. Díaz-Ramos L. A. Morales L. O. Strid Å. Jenkins G. I. (2019). Regulation of Arabidopsis gene expression by low fluence rate UV-B independently of UVR8 and stress signaling. Photoch. Photobio. Sci. 18, 1675–1684. doi: 10.1039/c9pp00151d 31218318

[B60] Pant S. Huang Y. (2022). Genome-wide studies of *PAL* genes in sorghum and their responses to aphid infestation. Sci. Rep. 12, 22537. doi: 10.1038/s41598-022-25214-1 36581623 PMC9800386

[B61] Pfeiffer B. K. Rooney W. L. (2015). Sunlight induces black color and increases flavonoid levels in the grain of sorghum line Tx3362. Crop Sci. 55, 1703–1711. doi: 10.2135/cropsci2014.11.0757

[B62] Pfeiffer B. K. Rooney W. L. (2016). Inheritance of pericarp color, nutritional quality, and grain composition traits in black sorghum. Crop Sci. 56, 164–172. doi: 10.2135/cropsci2015.04.0224

[B63] Pourcel L. Irani N. G. Lu Y. Riedl K. Schwartz S. Grotewold E. (2010). The formation of anthocyanic vacuolar inclusions in *Arabidopsis thaliana* and implications for the sequestration of anthocyanin pigments. Mol. Plant 3, 78–90. doi: 10.1093/mp/ssp071 20085894 PMC2807924

[B64] Raina S. K. Wankhede D. Sinha A. (2013). *Catharanthus roseus* mitogen-activated protein kinase 3 confers UV and heat tolerance to *Saccharomyces cerevisiae* . Plant Signal Behav. 8, e22716. doi: 10.4161/psb.22716 23221751 PMC3745576

[B65] Rao X. Dixon R. A. (2019). Co-expression networks for plant biology: Why and how. Acta Bioch. Bioph. Sin. 51, 981–988. doi: 10.1093/abbs/gmz080 31436787

[B66] Rejeb I. B. Pastor V. Mauch-Mani B. (2014). Plant responses to simultaneous biotic and abiotic stress: Molecular mechanisms. Plants (Basel). 3, 458–475. doi: 10.3390/plants3040458 27135514 PMC4844285

[B67] Riaz B. Chen H. Wang J. Du L. Wang K. Ye X. (2019). Overexpression of maize *Zm*C1 and *Zm*R transcription factors in wheat regulates anthocyanin biosynthesis in a tissue-specific manner. Int. J. Mol. Sci. 20, 5806. doi: 10.3390/ijms20225806 31752300 PMC6887777

[B68] Sacco A. Raiola A. Calafiore R. Barone A. Rigano M. M. (2019). New insights in the control of antioxidants accumulation in tomato by transcriptomic analyses of genotypes exhibiting contrasting levels of fruit metabolites. BMC Genomics 20, 43. doi: 10.1186/s12864-019-5428-4 30646856 PMC6332538

[B69] Saed-Moucheshi A. Shekoofa A. Pessarakli M. (2014). Reactive oxygen species (ROS) generation and detoxifying in plants. J. Plant Nutr. 37, 1573–1585. doi: 10.1080/01904167.2013.868483

[B70] Sahu A. Singh R. Verma P. K. (2023). Plant transcription factors: Unlocking multilayered regulation in development, stress and immunity. Planta 258, 31. doi: 10.1007/s00425-023-04188-y 37368167

[B71] Shi C. Liu H. (2021). How plants protect themselves from ultraviolet-B radiation stress. Plant Physiol. 187, 1096–1103. doi: 10.1093/plphys/kiab245 34734275 PMC8566272

[B72] Shih C.-H. Chu I. K. Yip W. K. Lo C. (2006). Differential expression of two flavonoid 3′-hydroxylase cDNAs involved in biosynthesis of anthocyanin pigments and 3-deoxyanthocyanidin phytoalexins in sorghum. Plant Cell Physiol. 47, 1412–1419. doi: 10.1093/pcp/pcl003 16943219

[B73] Snyder B. A. Nicholson R. L. (1990). Synthesis of phytoalexins in sorghum as a site-specific response to fungal ingress. Science 248, 1637–1639. doi: 10.1126/science.248.4963.1637 17746504

[B74] Stalikas C. D. (2007). Extraction, separation, and detection methods for phenolic acids and flavonoids. J. Sep. Sci. 30, 3268–3295. doi: 10.1002/jssc.200700261 18069740

[B75] Strid Å. Chow W. S. Anderson J. M. (1994). UV-B damage and protection at the molecular-level in plants. Photosynth. Res. 39, 475–489. doi: 10.1007/Bf00014600 24311138

[B76] Tang D. Wang G. Zhou J. M. (2017). Receptor kinases in plant-pathogen interactions: More than pattern recognition. Plant Cell 29, 618–637. doi: 10.1105/tpc.16.00891 28302675 PMC5435430

[B77] Tello-Ruiz M. K. Jaiswal P. Ware D. (2022). Gramene: A resource for comparative analysis of plants genomes and pathways. Methods Mol. Biol. 2443, 101–131. doi: 10.1007/978-1-0716-2067-0_5 35037202

[B78] Tiku A. R. (2020). “Antimicrobial compounds (phytoanticipins and phytoalexins) and their role in plant defense,” in Co-Evolution of Secondary Metabolites. Reference Series in Phytochemistry. Eds. Mérillon J. M. Ramawat K. (Springer, Cham), 845–868.

[B79] Tugizimana F. Djami-Tchatchou A. T. Steenkamp P. A. Piater L. A. Dubery I. A. (2019). Metabolomic analysis of defense-related reprogramming in *Sorghum bicolor* in response to *Colletotrichum sublineolum* infection reveals a functional metabolic web of phenylpropanoid and flavonoid pathways. Front. Plant Sci. 9. doi: 10.3389/fpls.2018.01840 PMC6328496 30662445

[B80] van Loon L. C. Rep M. Pieterse C. M. (2006). Significance of inducible defense-related proteins in infected plants. Annu. Rev. Phytopathol. 44, 135–162. doi: 10.1146/annurev.phyto.44.070505.143425 16602946

[B81] Wu Y. Li X. Xiang W. Zhu C. Lin Z. Wu Y.. (2012). Presence of tannins in sorghum grains is conditioned by different natural alleles of *tannin1* . Proc. Natl. Acad. Sci. U.S.A. 109, 10281–10286. doi: 10.1073/pnas.1201700109 22699509 PMC3387071

[B82] Wu J. Mohamed D. Dowhanik S. Petrella R. Gregis V. Li J.. (2020). Spatiotemporal restriction of *FUSCA*3 expression by Class I BPCS promotes ovule development and coordinates embryo and endosperm growth. Plant Cell. 32, 1886–1904. doi: 10.1105/tpc.19.00764 32265266 PMC7268797

[B83] Wu Y. B. Wang Y. L. Liu Z. Y. Wang J. (2023). Extraction, identification and antioxidant activity of 3-deoxyanthocyanidins from *Sorghum bicolor* L. Moench cultivated in China. Antioxidants-Basel 12, 468. doi: 10.3390/antiox12020468 36830026 PMC9952376

[B84] Xu J. Wang W. Zhao Y. (2021). Phenolic compounds in whole grain sorghum and their health benefits. Foods 10, 1921. doi: 10.3390/foods10081921 34441697 PMC8392263

[B85] Yamaguchi Y. Huffaker A. (2011). Endogenous peptide elicitors in higher plants. Curr. Opin. Plant Biol. 14, 351–357. doi: 10.1016/j.pbi.2011.05.001 21636314

[B86] Yan J. Liu Y. Yang L. He H. Huang Y. Fang L.. (2021). Cell wall beta-1,4-galactan regulated by the BPC1/BPC2-GALS1 module aggravates salt sensitivity in *Arabidopsis thaliana* . Mol. Plant 14, 411–425. doi: 10.1016/j.molp.2020.11.023 33276159

[B87] Yang L. Browning J. D. Awika J. M. (2009). Sorghum 3-deoxyanthocyanins possess strong phase II enzyme inducer activity and cancer cell growth inhibition properties. J. Agr. Food Chem. 57, 1797–1804. doi: 10.1021/jf8035066 19256554

[B88] Yang X. Wang J. Xia X. Zhang Z. He J. Nong B.. (2021). *Osttg1*, a WD40 repeat gene, regulates anthocyanin biosynthesis in rice. Plant J. 107, 198–214. doi: 10.1111/tpj.15285 33884679

[B89] Zhang S. Y. Chen Y. X. Zhao L. L. Li C. Q. Yu J. Y. Li T. T.. (2020). A novel NAC transcription factor, *Md*NAC42, regulates anthocyanin accumulation in red-fleshed apple by interacting with *Md*MYB10. Tree Physiol. 40, 413–423. doi: 10.1093/treephys/tpaa004 32031661

[B90] Zhou Y. Lv J. Yu Z. Wang Z. Li Y. Li M.. (2022). Integrated metabolomics and transcriptomic analysis of the flavonoid regulatory networks in *Sorghum bicolor* seeds. BMC Genomics 23, 619. doi: 10.1186/s12864-022-08852-7 36028813 PMC9414139

[B91] Zhuang W. B. Li Y. H. Shu X. C. Pu Y. T. Wang X. J. Wang T.. (2023). The classification, molecular structure and biological biosynthesis of flavonoids, and their roles in biotic and abiotic stresses. Molecules 28, 3599. doi: 10.3390/molecules28083599 37110833 PMC10147097

