# Supplementary material for: UV-induced reactive oxygen species and transcriptional control of 3-deoxyanthocyanidin biosynthesis in black sorghum pericarp
Source: Front Plant Sci. 2024 Oct 7;15:1451215. doi: 10.3389/fpls.2024.1451215 (PMC11491397; doi:10.3389/fpls.2024.1451215)
Supplement: Supplementary file 3 [file Table1.pdf]

Supplementary Material

Supplementary Table 1. Two-way ANOVA results of H_2O_2 concentrations (μmol·mg FW^{-1}) in pericarp tissue of genotypes BTx378 and RTx3362 at 5, 10, and 17 days after anthesis (DAA) under full sunlight and shaded conditions.

SOV[a]	DF[b]	SS[c]	MS[d]	F value	P(>F)[e]
**Genotype**	1	1.29E-09	1.29E-09	126.09	0.01068
**Light Treatment**	1	2.33E-11	2.33E-11	2.27152	0.28177
**DAA**	2	3.39E-11	1.70E-11	1.65293	0.38909
**DAA*Light Treatment**	2	5.34E-11	2.67E-11	2.59823	0.29212
**Genotype*Light Treatment**	1	2.35E-10	2.35E-10	22.9283	0.04865
**Genotype*DAA**	2	1.43E-10	7.17E-11	6.97961	0.13932
**Error**	26	2.55E-10	9.81E-12		

[a] source of variation
[b] degrees of freedom
[c] sum of squares
[d] mean squares
[e] Prob > F
