# Supplementary material for: UV-induced reactive oxygen species and transcriptional control of 3-deoxyanthocyanidin biosynthesis in black sorghum pericarp
Source: Front Plant Sci. 2024 Oct 7;15:1451215. doi: 10.3389/fpls.2024.1451215 (PMC11491397; doi:10.3389/fpls.2024.1451215)
Supplement: Supplementary file 4 [file Table2.pdf]

Supplementary Material

Supplementary Table 2. Complete list of hub genes in the Darkgrey WGCNA module from the combined analysis displaying correlations to pericarp LUT levels (G.S.LUT) and the number of gene connections at weight threshold > 0.2.

Gene Name	Gene Description[a]	Function[b]	G.S.LUT	Connections
**Sobic.005G136300**	CHS WHP1	Flavonoid biosynthesis	0.95	50
**Sobic.002G000400**	FSNII	Flavonoid biosynthesis	0.95	49
**Sobic.006G226800**	FNR, *P* gene	Flavonoid biosynthesis	0.92	32
**Sobic.009G043800**	DFR	Flavonoid biosynthesis	0.90	21
**Sobic.004G200788**	F3'H	Flavonoid biosynthesis	0.83	15
**Sobic.005G137200**	CHS WHP1	Flavonoid biosynthesis	0.55	14
**Sobic.004G200744**	F3'H	Flavonoid biosynthesis	0.77	33
**Sobic.004G050200**	DFR	Flavonoid biosynthesis	0.92	10
**Sobic.005G137133**	CHS	Flavonoid biosynthesis	0.49	10
**Sobic.010G230900**	Cytochrome P450 CYP2	Secondary metabolism	0.99	48
**Sobic.004G190800**	Galactosyltransferase	Secondary metabolism	0.93	42
**Sobic.010G231000**	*O*-Methyltransferase	Secondary metabolism	0.99	42
**Sobic.007G141200**	Cinnamoyl-CoA reductase	Secondary metabolism	0.94	41
**Sobic.007G058800**	*O*-Methyltransferase	Secondary metabolism	0.95	31
**Sobic.007G223900**	UDP-glucosyl transferase	Secondary metabolism	0.72	29
**Sobic.004G304200**	short chain dehydrogenase/reductase	Secondary metabolism	0.93	28
**Sobic.010G238400**	*O*-glucosyltransferase	Secondary metabolism	0.94	23
**Sobic.010G259300**	Benzyl alcohol *O*-benzoyltransferase	Secondary metabolism	0.51	7
**Sobic.010G193000**	Unknown function	Secondary metabolism	0.43	1
**Sobic.010G230800**	*O*-Methyltransferase ZRP4	Secondary metabolism	0.29	1
**Sobic.001G071000**	NAC Domain	TF	0.95	45
**Sobic.003G031100**	bHLH	TF	0.97	41
**Sobic.010G035300**	WRKY72	TF	0.90	36
**Sobic.003G349600**	C2H2-type zinc finger	TF	0.88	35
**Sobic.008G183900**	NAC Domain	TF	0.88	29

**Sobic.009G164600**	C2H2-type zinc finger	TF	0.60	18
**Sobic.009G234100**	WRKY	TF	0.73	1
**Sobic.003G216000**	ABC transporter	Metabolic transport	0.93	39
**Sobic.001G136300**	Nodulin-like family protein	Metabolic transport	0.95	31
**Sobic.005G212700**	GST	Metabolic transport	0.85	19
**Sobic.003G216232**	ABC transporter	Metabolic transport	0.72	19
**Sobic.006G178500**	Amino acid transporter	Metabolic transport	0.76	19
**Sobic.004G087700**	pleiotropic drug resistance protein 7	Metabolic transport	0.58	1
**Sobic.001G317200**	GST	Metabolic transport	0.48	1
**Sobic.010G004200**	EamA-like transporter	Metabolic transport	0.91	13
**Sobic.009G012900**	LRR	Cellular signaling	0.97	43
**Sobic.001G074800**	Receptor-type protein kinase LRK1	Cellular signaling	0.86	22
**Sobic.005G126200**	Brassinosteroid-insensitive associated receptor kinase glycoprotein precursor	Cellular signaling	0.87	19
**Sobic.002G024100**	Lectin-domain receptor-like kinase	Cellular signaling	0.84	18
**Sobic.002G024300**	Lectin-domain receptor-like kinase	Cellular signaling	0.85	15
**Sobic.006G208300**	Auxin induced like protein	Cellular signaling	0.85	12
**Sobic.006G229600**	Lectin-domain receptor-like kinase	Cellular signaling	0.78	2
**Sobic.002G024000**	Serine/threonine protein kinase	Cellular signaling	0.65	6
**Sobic.001G401300**	Pathogenesis-related protein 10a	Stress defense response	0.88	56
**Sobic.005G101600**	Nucleoporin-related // Dirigent protein	Stress defense response	0.89	45
**Sobic.005G101800**	Nucleoporin-related // Dirigent protein	Stress defense response	0.92	37
**Sobic.005G101500**	Nucleoporin-related // Dirigent protein	Stress defense response	0.81	28
**Sobic.003G111200**	Potato type II proteinase inhibitor family	Stress defense response	0.93	28
**Sobic.005G169200**	Pathogenesis-related protein 4	Stress defense response	0.81	25
**Sobic.008G182700**	Pathogenesis-related protein 4	Stress defense response	0.69	23
**Sobic.008G182900**	Pathogenesis related protein-5	Stress defense response	0.90	23
**Sobic.003G111300**	serine type endopeptidase inhibitor	Stress defense response	0.87	22
**Sobic.005G169400**	Pathogenesis-related protein	Stress defense response	0.76	18
**Sobic.008G182800**	Thaumatin-like pathogenesis-related protein 4 precursor	Stress defense response	0.72	12
**Sobic.005G166200**	MTN26L5 - MtN26 family protein precursor	Stress defense response	0.44	11

**Sobic.003G413700**	potassium transporter	Stress defense response	0.80	10
**Sobic.010G020200**	Pathogenesis-related protein PRMS precursor	Stress defense response	0.43	1
**Sobic.008G182600**	Pathogenesis related protein-5	Stress defense response	0.54	4
**Sobic.005G101700**	Dirigent-like protein	Stress defense response	0.82	19
**Sobic.001G401100**	Pathogenesis-related protein 10a	Stress defense response	0.59	2
**Sobic.007G011700**	Bile acid beta-glucosidase	Stress defense response	0.88	20
**Sobic.005G215900**	Bowman-Birk serine protease inhibitor	Stress defense response	0.47	2
**Sobic.005G216000**	Proteinase inhibitor I12, Bowman-Birk	Stress defense response	0.49	13
**Sobic.003G422200**	Glucan endo-1,3-beta-D-glucosidase / Laminarinase	Stress defense response	0.60	19
**Sobic.002G074400**	NAD(P)H:quinone oxidoreductase, WrbA	Redox homeostasis	0.90	25
**Sobic.002G004700**	NAD(P)H:quinone oxidoreductase, WrbA	Redox homeostasis	0.78	13
**Sobic.001G264000**	Alcohol dehydrogenase 2	Redox homeostasis	0.86	12
**Sobic.004G304300**	short-chain dehydrogenase/reductase	Redox homeostasis	0.82	9
**Sobic.005G082700**	Oxidoreductase, zinc-binding dehydrogenase family	Redox homeostasis	0.69	1
**Sobic.002G024400**	serine carboxypeptidase II	Miscellaneous	0.79	4
**Sobic.005G137400**	Unknown function	Miscellaneous	0.91	31
**Sobic.001G451500**	Glutamine synthetase root isozyme 1	Miscellaneous	0.91	38
**Sobic.004G191300**	Unknown function	Miscellaneous	0.89	29
**Sobic.010G042300**	Os06g0152700 protein	Miscellaneous	0.82	22
**Sobic.003G278000**	Unknown function	Miscellaneous	0.70	16
**Sobic.003G203200**	Unknown function	Miscellaneous	0.80	13
**Sobic.003G111900**	Laccase LAC2-1	Miscellaneous	0.44	10
**Sobic.001G453100**	Homocysteine S-methyltransferase 1	Miscellaneous	0.71	13
**Sobic.004G165200**	Hydrolase, alpha/beta fold protein-like	Miscellaneous	0.96	33
**Sobic.001G400800**	Bet v I/Major latex protein // START-like domain // Bet v I type allergen	Miscellaneous	0.72	30
**Sobic.001G400700**	Bet v I/Major latex protein // START-like domain // Bet v I type allergen	Miscellaneous	0.59	13
**Sobic.006G273600**	H0913C04.5 protein	Miscellaneous	0.83	29
**Sobic.001G401800**	Unknown function	Miscellaneous	0.62	8

[a] Gene description obtained from Phytozome description based on Sbicolor_730_v5.0 (https:// phytozome- next.jgi.doe.gov/info/Sbicolor_v5_1) reference genome.

[b] Gene function obtained from literature search.
